# ZnCl_2_-Based Deep Eutectic Solvent as Solvent-Catalyst in the Michael Addition Reaction of Pyrrole to Maleimide

**DOI:** 10.3390/molecules29225381

**Published:** 2024-11-15

**Authors:** Abelardo Gutiérrez-Hernández, Fátima M. Soto-Suárez, Arlette Richaud, Francisco Méndez, Claudia Araceli Contreras-Celedón

**Affiliations:** 1 Departamento de Síntesis Orgánica, Instituto de Investigaciones Químico Biológicas, Universidad Michoacana de San Nicolás de Hidalgo, Morelia 58030, Mexicofatima.soto@umich.mx (F.M.S.-S.); 2 CEMHTI-CNRS, 45100 Orléans, France; arletterichaud@gmail.com; 3 Departamento de Química, División de Ciencias Básicas e Ingeniería, Universidad Autónoma Metropolitana-Iztapalapa, Ciudad de México 09340, Mexico

**Keywords:** DESs, Lewis acid, Michael addition, hydrogen bond, zinc-zincate species 2ZnCl_2_·3H_2_O

## Abstract

The use of deep eutectic solvents (DESs) as catalysts presents indisputable advantages, for example, their simplicity of preparation, high biodegradability, and recyclability, as well as zero toxicity and their effectiveness as environmentally friendly reaction media. However, aspects related to their reactivity and catalytic activity are still unclear. In this work, we explore the versatility of ChCl/ZnCl_2_ DES in the formation of C-C bonds through the Michael-type addition of pyrrole to maleimide, where ChCl/ZnCl_2_ DES leads to catalysis and chelation of the substrates, thus describing a recommended method for the construction of C-C bonds with high atomic economy. We describe experimental and theoretical aspects that explain the ability of ChCl/ZnCl_2_ DES in the presence of water to act as a catalyst in the formation of C-C bonds between pyrrole and maleimide. The potential energy surface showed that the ChCl and the zinc-zincate species 2ZnCl_2_·3H_2_O, formed by the interaction between zinc chloride and water, decrease the relative free Gibbs energy values for all the species involved in the reaction mechanism (TSs, intermediates, product), favoring the kinetics and thermodynamics of the Michael addition.

## 1. Introduction

Catalysis plays a fundamental role in obtaining the vast majority of chemical products used today. The use of catalysts has allowed for an acceleration of the population of the chemical space [1], originating a wide variety of new organic compounds by increasing the efficiency and selectivity of chemical reactions.

In organic synthesis, the use of transition metals was a turning point in catalysis research, exponentially accelerating the discovery of new synthetic routes that were previously unthinkable and facilitating processes such as the formation of C-C bonds, which, being ubiquitous in organic compounds, often become a crucial step when considering the synthesis of any of them. Although the formation of C-C bonds can be carried out with high efficiency by using transition metals such as Rh, Ru, Ir, Pd, and Co [2,3,4,5,6], they generate toxic waste [7,8], in addition to having an increasingly high cost due to the overexploitation of non-renewable resources.

In recent years, there has been a change in the focus of catalysis research, seeking to align with the principles of green chemistry [9] and prioritizing the use of environmentally friendly resources and processes. In this context, solvents have been considered as a strategic sector to mitigate the environmental impact generated by the chemical industry [10]; thus, a series of new non-volatile alternative solvents has emerged, among which the deep eutectic solvents (DESs) stand out, which have gained popularity within the last decade due to their usefulness in multiple applications in electrochemistry [11,12], CO_2_ capture [13], extraction of natural products [14], organic synthesis [15], and catalysis [16]. All this is due to their unique properties, such as good thermal stability, low vapor pressure, no or low toxicity, easy preparation with high atomic economy, and excellent environmental compatibility.

The application of DESs as reaction media is well known; however, their catalytic activity has received little attention, and even less has been devoted to studying the mechanism of the catalytic activity they present [17]. Among the advantages of using DESs as catalysts is the possibility of using them in stoichiometric quantities. They present a greater catalytic effect than any of their components alone, in addition to their reusability without significant loss of their catalytic activity, making them an attractive alternative to conventional catalysts.

DESs are formed by combining molecules with hydrogen bond donor (HBD) and hydrogen bond acceptor (HBA) characteristics. Acidic molecules of the Lewis or Brønsted types can be included as HBDs, and quaternary ammonium salts are commonly used as HBAs. Knowing that the components of DESs form hydrogen bonds between them, it is important to consider that the acidic characteristics of DESs facilitate the breaking and formation of bonds in the organic reactions where they are used. The possibility of having numerous mixtures of HBD and HBA favors the synthesis of DESs with physicochemical properties determined by the nature of their components and the type of interaction between them [18,19], which suggests that a suitable DES could be synthesized for each type of reaction [20,21].

Zinc is not only one of the chemical elements capable of performing important biological functions, but it is also involved in various chemical processes. This is due to its Lewis acid nature, which favors higher substrate reactivity, selectivity, and mild reaction conditions. Therefore, ZnCl_2_ has been used in the formation of Lewis acid deep eutectic solvents (LADESs), which have been used as solvents and catalysts in various chemical reactions, for example, in the acylation reactions of anilines [22], synthesis of indoles [23], esterification of carboxylic acids [24], and Friedel–Crafts alkylations [25]. The findings of Zhao in 2013 are particularly pertinent to our research, as they describe the Michael addition of pyrroles to maleimide in the presence of ZnCl_2_ or AlCl_3_ catalysts, using 1,2-dichloroethane at reflux [26].

In a previous study conducted by our research group, the catalytic role of the ChCl/*p*-TsOH DES (1:1) in an aza-Michael conjugate addition reaction of anilines to maleimides was described. It was observed that the DES acted as a stabilizer of the transition states involved in bond formation, which were favored by enthalpy [27]. We now proceed to present the findings of our experimental and theoretical investigations into the catalytic effect of ChCl/ZnCl_2_ DES (1:2) in the reaction of C-C bond formation between pyrrole **1** and maleimides **2**.

## 2. Results and Discussion

In this study, we conducted both experimental and theoretical examinations of the impact of ZnCl_2_ as the acidic component of DES on the conjugated addition reaction of pyrrole **1** and maleimides **2**. To achieve this objective, four distinct DESs were synthesized, utilizing ZnCl_2_ as the hydrogen bond donor and ChCl, urea, D-glucose, and D-(-)-fructose as hydrogen bond acceptors, respectively. The synthesis procedures used for each of the DES followed the methodologies described in the literature [28,29].

The experimental study began with the analysis of the reaction between compounds **1** and **2a** in presence of ChCl/ZnCl_2_ DES at a temperature of 100 °C. After two hours, the decomposition of pyrrole was observed, and the formation of product **3a** was not detected. However, the C-C bond between pyrrole **1** and maleimides **2** was achieved at room temperature using different DESs, such as ChCl/ZnCl_2_, urea/ZnCl_2_, glucose/ZnCl_2_, and fructose/ZnCl_2_, and the highest yields were observed with the ChCl/ZnCl_2_ DES (Table 1).

**Scheme 1:**
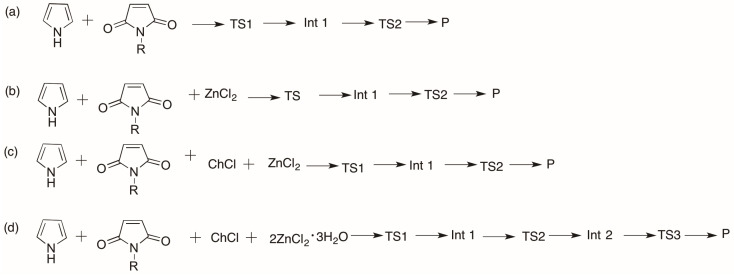
Set of reactions showing the mechanisms involved in the Michael addition between pyrrole **1** and maleimides **2a** and **2b** with and without DES ChCl/ZnCl_2_. TS1= transition state 1, Int1 = intermediary 1, TS2 = transition state 2, Int2 = intermediary 2, TS3 = transition state 3, P = product.

As we can observe, the yield of the synthesis of **3a** and **3b** increased when DES based on ChCl/ZnCl_2_ was used, which leads us to think about the role of ChCl and ZnCl_2_ in the formation of the C-C bond between pyrrole **1** and maleimides **2a** and **2b**. Therefore, we proposed a reaction mechanism for the Michael addition of the reagents pyrrole **1** and maleimides **2a** and **2b** (entry **a**/Scheme 1), and then incorporated ZnCl_2_ (entry **b**/Scheme 1), ChCl/ZnCl_2_ DES (entry **c**/Scheme 1), and ChCl + 2ZnCl_2_·3H_2_O (entry **d**/Scheme 1).

We obtained the potential energy surface for the reaction mechanisms proposed for the reagents pyrrole **1** and maleimides **2a** and **2b** with and without ChCl/ZnCl_2_ DES. As rates and equilibrium are related to Gibbs free energy, we calculated the *G*^0^ values of the species involved in the reactions at the PM6 level of theory using Gaussian 09 program [30]. Nakata and Maeba obtained good correlations between data obtained using the B3LYP/6-31G*//PM6 and PM6//PM6 methods and concluded that “…These comprehensive results pave the way for applications in drug discovery and materials science, among others…” [31]. Table 2 shows the relative Gibbs free energy values for all of the species involved in the reaction mechanisms. They refer Gibbs free energy of the reagents.

**Figure 1:**
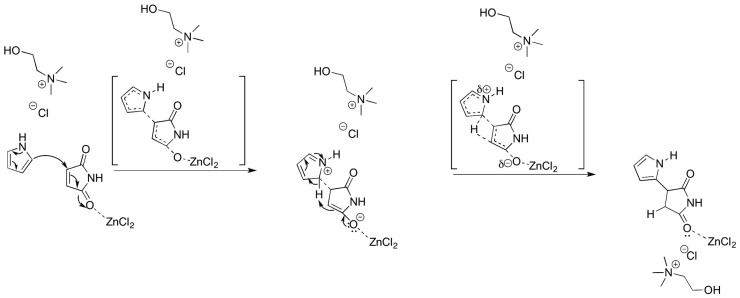
Reaction mechanism for the addition of pyrrole **1** to maleimide **2a** in the presence of ChCl/ZnCl_2_ DES.

Table 2 shows that the interaction between pyrrole **1** and maleimides **2a** and **2b** was unfavorable (reaction mechanism **a**). The formation of TS1, Intermediate 1, and TS2 occurred with the highest values of Gibbs free energy (relative to reagents). However, the formation of the product was favored. The ${∆G}_{r}^{0}$ values were −22.76 kcal/mol for maleimide **2a** and −21.48 kcal/mol for N-phenylmaleimide **2b**, and both reactions were spontaneous (exergonic). Reaction mechanisms **b** and **c** show that the relative Gibbs free energy values decreased, and the Michael addition in the presence of ZnCl_2_ and ChCl/ZnCl_2_ DES became more favorable than the previous one. Furthermore, the formation of the intermediate 1 and product (relative to reagents) in the presence of ChCl/ZnCl_2_ DES was spontaneous, and the ${∆G}_{1}^{0}$ and ${∆G}_{r}^{0}$ values became negative −20.93 (−15.32) and −37.68 (−34.90) kcal/mol, respectively). Figure 1 shows that the choline chloride acted as a bond hydrogen acceptor, with the pyrrole **1** increasing the nucleophilic character of the C-2 carbon atom, while the zinc chloride behaved as a Lewis acid that interacted with the oxygen atom of maleimide **2a**, increasing the electrophilic property of the olefinic carbon.

**Figure 2:**
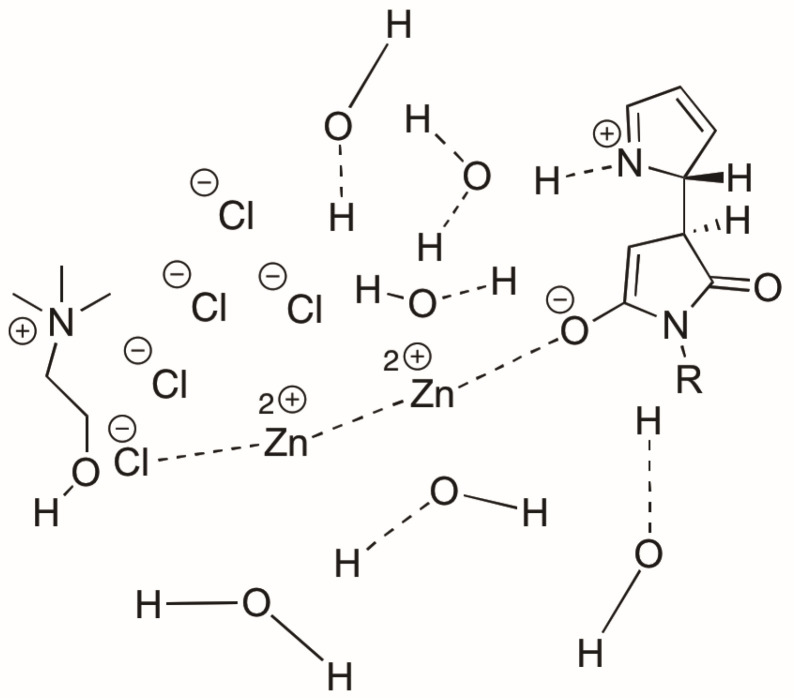
Schematic view of intermediate 1 for the reaction between pyrrole, maleimide, ChCl, and the zinc zincate species.

Wilcox et al. demonstrated that zinc-zincate species are formed through the hydration of ZnCl_2_ [32]. In our experiments, the ZnCl_2_ which was utilized was hydrated, and 0.1 mL of H_2_O was added to the mixture of reaction (see Table 1). Therefore, we included the zinc-zincate species 2ZnCl_2_·3H_2_O in the Michael addition reaction mechanism (**d** in Scheme 1). Entry **d**/Table 2 shows that the ChCl and the zinc-zincate species 2ZnCl_2_·3H_2_O decreased the free Gibbs energy values for all the species (relative to reagents) favoring the kinetics and thermodynamics of the Michael addition. The values for all the species were lower than those of the reagents, except for TS1 and TS2, which were 11.40 (13.62) and 6.38 (5.18) kcal/mol higher than those of the reagents, respectively. In the zinc-zincate species, six water molecules interacted with one Zn (II) in octahedral geometry and four chloride anions bound to the other Zn(II) in tetrahedral geometry. Figure 2 shows (in schematic view) how five chloride anions formed several hydrogen bonds with the choline atoms, while the six water molecules maintained the pyrrole and maleimide reagents in the correct geometric arrangement to obtain the maximum interaction between them with the lowest energy cost.

**Figure 3:**
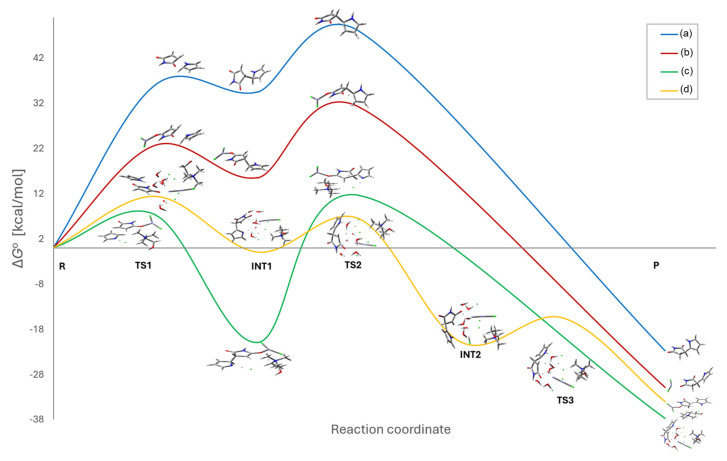
Gibbs energy profiles for the addition of pyrrole **1** to maleimide **2a** in the absence and presence of ZnCl_2_, ChCl/ZnCl_2_ DES, and zinc-zincate species 2ZnCl_2_·3H_2_O. (a), (b), (c), and (d) are the set of reaction mechanisms involved in the Michael addition in this reaction.

**Figure 4:**
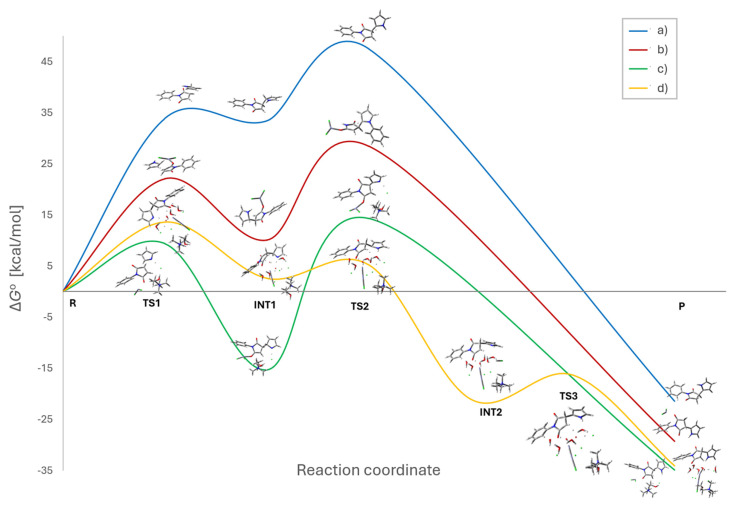
Gibbs energy profiles for the addition of pyrrole **1** to maleimide **2b** in the absence and presence of ZnCl_2_, ChCl/ZnCl_2_ DES, and zinc−zincate species 2ZnCl_2_·3H_2_O. (a), (b), (c), and (d) are the set of reaction mechanisms involved in the Michael addition in this reaction.

Figure 3 and Figure 4 show, respectively, the energy profiles for the set of reactions involved in the Michael addition between pyrrole **1** and maleimides **2a** and **2b,** with and without DES CHCl/ZnCl_2_ (see Scheme 1). The blue, red, yellow, and green lines display the addition of the reagents pyrrole **1** and maleimides **2a** and **2b**, as well as the presence of ZnCl_2_, ChCl/ZnCl_2_ DES, and ChCl + 2ZnCl_2_·3H_2_O, respectively. The potential energy surfaces clearly demonstrate how the zinc chloride, ChCl/ZnCl_2_ DES, and ChCl + 2ZnCl_2_·3H_2_O favored the kinetic and thermodynamic parameters for the formation of the C-C bond between pyrrole **1** and maleimides **2a** and **2b**.

## 3. Materials and Methods

### 3.1. Experimental

Thin-layer chromatography (TLC) analyses were performed on commercial aluminum plates bearing a 0.25 mm Merck silica gel 60F_254_ layer, which were visualized with UV light at 254 nm or under iodine. Column chromatography was performed with SiO_2_ (F60 (230–400 mesh)). Infra-red (IR) spectra were recorded on a Nicolet spectrometer, Thermo Scientific model iS10 (China) Ltd. (Shanghai, China) using ATR (Attenuated Total Reflection). Selected absorption maxima (ν_max_) are reported in wavenumbers (cm^−1^). Melting points were recorded in degrees Celsius (°C) using a Fisher–Johns melting point apparatus, and are reported uncorrected. ^1^H and ^13^C-NMR spectra of the solutions in CD_3_OD were recorded on a Varian Mercury (400 MHz) NMR spectrometer (Varian Inc., Palo Alto, CA, USA). Deuterated chloroform was used as received, and chemical shift values (δ) are reported in parts per million (ppm) relative to the residual signals of this solvent [δ 7.2 for ^1^H (CDCl_3_) and δ 77.2 ppm for ^13^C (CDCl_3_)]. Abbreviations used in the NMR follow-up experiments are as follows: *s*, singlet; *d*, doublet; *t*, triplet, and *m*, multiplet. Pyrrole **1**, maleimides **2**, ChCl, ZnCl_2,_ urea, D-glucose, and D-(-)-fructose were obtained from commercial sources and used as received.

#### 3.1.1. Preparation of Deep Eutectic Solvent (ChCl:ZnCl_2_)

First, 1.24 mmol of choline chloride and 2.5 mmol of ZnCl_2_ were mixed in a 5 mL round-bottomed flask and heated for 15 min at 120 °C until a clear liquid appeared. The mixture was stirred for 15 min more, and the colorless liquid was used directly for the reactions without purification.

#### 3.1.2. General Procedure for the Pyrrole–Maleimide Michael Addition in ChCl:ZnCl_2_

Next, 0.1 mL of water was added to the ChCl:ZnCl_2_ (0.650 g) with stirring for 5 min, and then the maleimide **2a** (0.072 g, 0.74 mmol, 1 equiv) and pyrrole **1** (0.05 g, 0.74 mmol, 1 equiv) were slowly added to the reaction mixture. The reaction mixture was stirred at room temperature for 2.5 h. TLC monitored the reaction. After completion of the reaction, water was added, the extraction was performed with EtOAc, the organic phase was separated and dried on anhydrous Na_2_SO_4_, and the solvent was evaporated. The pure **3a** product was purified on a silica gel chromatographic column using the mixture of Hex/EtOAc (7:3, *v*/*v*) as the eluent.

#### 3.1.3. Characterization Data of Compounds

3-(1H-pyrrol-2-yl)pyrrolidine-2,5-dione (**3a**). According to the general procedure, an 82% yield (0.102 g) was obtained. Brown crystals, rf 0.28 (Hex/EtOAc 6:4), melting point (measured): 105–108 °C; ^1^H NMR (400 MHz, CD_3_OD; (Ch_3_)_4_Si) δ_H_ = 10.32 (s, 1H), 6.71 (td, *J* = 2.7, 1.4 Hz, 3H), 6.04 (dd, *J* = 5.5, 2.8 Hz, 1H), 5.99 (dd, *J* = 2.4, 1.7 Hz, 1H), 4.15 (dd, *J* = 9.5, 5.3 Hz, 1H), 3.14 (dd, *J* = 18.2, 9.5 Hz, 1H), 2.82 (dd, *J* = 18.2, 5.3 Hz, 1H); ^13^C {^1^H} NMR (100 MHz, CD_3_OD; (Ch_3_)_4_Si) δ_C_ = 179.67, 178.62, 126.53, 118.22, 107.54, 105.49, 40.94, 36.72; IR (v/cm^−1^) 3352, 1769, 1682, 1188.

1-phenyl-3-(1H-pyrrol-2-yl)pyrrolidine-2,5-dione (**3b**) According to the general procedure, 3b was obtained in 87% yield (0.155 g). Colorless crystals, rf 0.35 (Hex/EtOAc 7:3), melting point (measured): 178–180 °C; ^1^H NMR (400 MHz, CDCl_3_, (Ch_3_)_4_Si) δH = 9.13 (s, 1H), 7.43–7.41 (m, 2H), 7.40–7.39 (ddd, J = 7.4, 3.7, 1.2 Hz, 1H), 7.29–7.26 (m, 2H), 6.83 (s, 1H), 6.22–6.20 (dd, J = 6.1, 2.8 Hz, 1H), 6.10–6.08 (m, 1H), 4.27–4.24 (dd, J = 9.3, 5.6 Hz, 1H), 3.40–3.33 (dd, J = 18.3, 9.3 Hz, 1H), 3.25–3.19 (dd, J = 18.3, 5.6 Hz, 1H); ^13^C{^1^H} NMR (100 MHz, CDCl_3_, (Ch_3_)_4_Si) δC = 176.94, 174.97, 131.78, 129.48, 129.08, 126.65, 125.41, 119.23, 108.71, 105.98, 38.71, 34.42; IR (v/cm^−1^) 3318, 2940, 1685, 1396.

### 3.2. Calculation Methods

The equilibrium geometries in the gas phase for the reagents pyrrole (**1**) and maleimide (**2a** and **2b**), the ChCl/ZnCl_2_ DES formed by ChCl and ZnCl_2_, the transition states (TS1-TS3), the intermediates (Int1 and Int2), and the products (**3a** and **3b**) were obtained at the PM6 level of theory using Gaussian 09 program (Revision B.01) [33]. For the zinc zincate, the formula of the trihydrated zinc chloride salt reported by Wilcox et al. [32] was used (2ZnCl_2_ and 6H_2_O). The frequency analysis was conducted for the optimized geometries of the species, verifying that all frequencies were positive and that only one negative frequency was obtained for the transition states. We used PM6 for all the calculations because (a) it generally has advantages over other semiempirical methods of providing more precise ${∆H}_{f}^{0}$, geometries, H-bonds, etc., [30]; (b) S Nakata et al. [31] found that PM6-optimized geometries for molecules are reasonably accurate compared to B3LYP/6-31G*-optimized geometries; and (c) S Nakata et al. [31] found some electronic properties calculated for a set of molecules containing C, H, O, and N atoms at the B3LYP/6-31G*//PM6 and PM6//PM6 levels of theory and found an exceptionally high correlation for the two calculation methods based on linear regression analysis (R^2^ = 0.907).

## 4. Conclusions

The best reaction conditions for the formation of a C-C bond between pyrrole **1** and maleimides **2a** and **2b** have been defined experimentally. We demonstrated that the presence of ChCl/ZnCl_2_ DES favored the formation of the C-C bond. The potential energy surface, obtained at the PM6 level of theory, allowed us to propose a general reaction mechanism of the Michael addition and showed that ChCl/ZnCl_2_ DES favors the kinetic and thermodynamic parameters of the reaction between pyrrole 1 and maleimides **2a** and **2b**. The presence of water in the ChCl/ZnCl_2_ DES formed the ChCl and the zinc-zincate species 2(ZnCl_2_·3H_2_O) that decreased the free Gibbs energy values for all the species involved in the reaction mechanism (relative to reagents), favoring the kinetics and thermodynamics of the Michael addition. The four chloride anions formed several hydrogen bonds with the choline atoms, while the water molecules maintained the pyrrole and maleimide molecules in the correct geometric arrangement to obtain the maximum interaction between them with the lowest energy cost. We are conducting further studies of this system at the ab initio level of theory. Experimental and theoretical studies at the ab initio level of theory for the Michael addition reaction of pyrrole **1** with a set of N-substituted maleimides (with electron-donating and electron-withdrawing substituents), including **2a** and **2b**, are underway in our laboratories and will be reported in due course.

## Figures and Tables

**Table 1:** Conjugated addition of pyrrole **1** to maleimides **2a** and **2b** at DES.

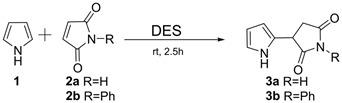				
Entry	DES	Reaction Temperature (°C)	3a ^a^ Yield (%) ^c^	3b ^b^ Yield (%) ^c^
1	ChCl/ZnCl_2_ (1:2)	100	---	---
2	ChCl/ZnCl_2_ (1:2)	25	82	87
3	Urea/ZnCl_2_ (3.5:1)	25	52	58
4	D-Glucosa/ZnCl_2_ (1:1)	25	11	16
5	D-(-)-Fructosa/ZnCl_2_ (1:1)	25	10	8

^a^ General conditions: pyrrole **1** 0.05 g (0.74 mmol, 1 equiv.), maleimide **2a** 0.072 g (0.74 mmol,1 equiv.), DES 0.650 g, 2.5 h, 0.1 mL of H_2_O was added. ^b^ General conditions: pyrrole **1** 0.05 g (0.74 mmol, 1 equiv.), with N-phenylmaleimide **2b** 0.129 g (0.74 mmol, 1 equiv.), DES 0.650 g, 2.5 h, 0.1 mL of H_2_O was added. ^c^ The yield was determined by gas–mass chromatography.

**Table 2:** Relative Gibbs free energy values for the species involved in the reaction mechanisms a–d. All the Δ*G*^0^ values are in kcal/mol and were obtained at T = 298.15 K and P = 1 atm. $G_{R}^{0}$ is the sum of the Gibbs free energies of the reagents.

Reaction Mechanism	$G_{TS1}^{0}-G_{R}^{0}$ $\left(∆G_{1}^{\neq }\right)$	$G_{Int1}^{0}-G_{R}^{0}$ $\left(∆G_{1}^{0}\right)$	$G_{TS2}^{0}-G_{R}^{0}$	${(G}_{Int2}^{0}-G_{R}^{0})$	${(G}_{TS3}^{0}-G_{R}^{0})$	$G_{P}^{0}-G_{R}^{0}$ $\left(∆G_{r}^{0}\right)$
a	36.06 ^i^ (33.87) ^ii^	34.47 (33.37)	47.92 (47.43)	-	-	−22.76 (−21.48)
b	22.67 (22.06)	15.51 (10.08)	31.15 (28.43)	-	-	−30.86 (−29.26)
c	7.32 (9.37)	−20.93 (−15.32)	11.61 (14.28)	-	-	−37.68 (−34.90)
d	11.40 (13.62)	−0.90 (2.58)	6.38 (5.18)	−20.86 (−21.08)	−15.47 (−16.35)	−34.00 (−34.08)

^i^ Michael addition of pyrrole **1** with maleimide **2a**. ^ii^ Michael addition of pyrrole **1** with N-phenylmaleimide **2b.**

## Data Availability

Data are contained within the article and Supplementary Materials.

## Supplementary Materials

The following supporting information can be downloaded at: https://www.mdpi.com/article/10.3390/molecules29225381/s1, Figure S1. ^1^H NMR (400 MHz, CD_3_OD), ^13^C{^1^H} NMR (100 MHz, CD_3_OD) *3-(1H-pyrrol-2-yl)pyrrolidine-2,5-dione)* **3a**, Figure S2. IR Spectrum 3-(1H-pyrrol-2-yl)pyrrolidine-2,5-dione **3a**, Figure S3. ^1^H NMR (400 MHz, CDCl_3_), ^13^C{^1^H} NMR (100 MHz, CDCl_3_) *1-phenyl-3-(1H-pyrrol-2-yl)pyrrolidine-2,5-dione* **3b**, Figure S4. IR Spectrum *1-phenyl-3-(1H-pyrrol-*2-yl)*pyrrolidine*-2,5-dione **3b**. Complete reference of Gaussian 09. Sum of electronic and zero-point energies, sum of electronic and thermal energies, sum of electronic and thermal enthalpies, sum of electronic and thermal free energies, values of molar heat capacities at constant volume, entropy and Cartesian coordinates of atoms (XYZ, in angstroms) for each molecule optimized at PM6 level of theory.
