# Supplementary material for: ZnCl2-Based Deep Eutectic Solvent as Solvent-Catalyst in the Michael Addition Reaction of Pyrrole to Maleimide
Source: Molecules. 2024 Nov 15;29(22):5381. doi: 10.3390/molecules29225381 (PMC11596833; doi:10.3390/molecules29225381)
Supplement: Supplementary file 1 [file molecules-29-05381-s001.zip › molecules-3300860-supplementary.pdf]

SUPPORTING INFORMATION

$ZnCl_2$-Based Deep Eutectic Solvent as Solvent-Catalyst in the Michael Addition Reaction of Pyrrole to Maleimide

Abelardo Gutiérrez-Hernández [1], Fátima M. Soto-Suárez [1], Arlette Richaud [2], Francisco Méndez [3,*] and Claudia Araceli Contreras-Celedón [1,*]

[1] Departamento de Síntesis Orgánica, Instituto de Investigaciones Químico Biológicas, Universidad Michoacana de San Nicolás de Hidalgo, Morelia 58030, Mexico

[2] CEMHTI-CNRS, 45100 Orléans, France

[3] Departamento de Química, División de Ciencias Básicas e Ingeniería, Universidad Autónoma Metropolitana-Iztapalapa, Ciudad de México 09340, Mexico

* Correspondence: fm@xanum.uam.mx (F.M.); claudia.contreras@umich.mx (C.A.C.-C.)

Índex

Tabla de contenido

Materials and equipment 4

Preparation of deep eutectic solvent (ChCl:$ZnCl_2$) 4

Typical procedure for the pyrrole-maleimide Michael addition in ChCl:$ZnCl_2$..... 4

3-(1H-pyrrol-2-yl)pyrrolidine-2,5-dione) 3a 5

1-phenyl-3-(1H-pyrrol-2-yl)pyrrolidine-2,5-dione 3b 5

Figure S1. ^1H NMR (400MHz, CD_3OD), $^{13}C\{^1H\}$ NMR (100 MHz, CD_3OD) 3-(1H-pyrrol-2-yl)pyrrolidine-2,5-dione) 3a 7

Figure S2. IR Spectrum 3-(1H-pyrrol-2-yl)pyrrolidine-2,5-dione 3a 7

Figure S3 ^1H NMR (400MHz, $CDCl_3$), $^{13}C\{^1H\}$ NMR (100 MHz, $CDCl_3$) 1-phenyl-3-(1h-pyrrol-2-yl)pyrrolidine-2,5-dione 3b 8

Figure S4. IR Spectrum1-phenyl-3-(1h-pyrrol-2-yl)pyrrolidine-2,5-dione 3b 9

Reference Gaussian 09 9

Theoretical calculations 10

Energy and coordinates of Pirrol 10

Energy and coordinates of Maleimide 10

Energy and coordinates of N-Phenyl Maleimide 11

Energy and coordinates of Choline chloride 12

Energy and coordinates of Zinc chloride 13

Energy and coordinates of Zinc zincate (2 $ZnCl_2$ • 3 H_2O) 14

Structures for the proposed reactions between Pirrol et Maleimide 15

Transition state a) TS1 15

Intermediate a) Int1 16

Transition state a)TS2 17

Succinimide a)P 18

Transition state b)TS1 19

Intermediate b)Int1 21

Transition state b)TS2 22

Succinimide + $ZnCl_2$ b) P 23
Transition state c)TS1 24
Intermediate c)Int1 26
Transition state c)TS2 28
Succinimide + ChCl + $ZnCl^2$ c)P 30
Transition state d)TS1 31
Intermediate d)Int1 34
Transition state d)TS2 36
Transition state d)TS3 39
Succinimide + ChCl + 2 $ZnCl_2$ • 3H2O d)P 41
Structures for the proposed reactions between Pirrol et N-Phenyl Maleimide 44
Transition state a) TS1 44
Intermediate a) Int1 45
Transition state a)TS2 47
N-Phenyl succinimide a)P 48
Transition state b) TS1 49
Intermediate b) Int1 51
Transition state b)TS2 53
N-Phenyl succinimide $ZnCl_2$ b)P 54
Transition state c) TS1 56
Intermediate c) Int1 58
Transition state c)TS2 60
Transition state d) TS1 64
Intermediate d) Int1 67
Transition state d)TS2 70
Intermediate b) Int2 72
Transition state b) TS3 75
N-Phenyl succinimide + ChCl + 2 $ZnCl_2$ • $3H_2O$ d)P 78

Experimental

Materials and equipment

Thin layer chromatography (TLC) analyses were performed on commercial aluminum plates bearing a 0.25 mm Merck silica gel 60F254 layer, visualized with UV light at 254 nm or under iodine. Column chromatography was performed with SiO_2 (F60 (230–400 mesh)). Infra-red (IR) spectra were recorded on a Thermo Scientific modelo Nicolet iS10 spectrometer using ATR (Attenuated Total Reflection). Selected absorption maxima (ν_{max}) are reported in wavenumbers (cm^{-1}). The melting point was recorded in degrees Celsius (°C), using a Fisher-Johns melting point apparatus, and is reported uncorrected. ^1H and ^{13}C-NMR spectra of solutions in CD_3OD were recorded on a Mercury 400 spectrometer. Deuterated chloroform was used as received, and chemical shift values (δ) are reported in parts per million (ppm) relatives to the residual signals of this solvent [δ 7.2 for ^1H ($CDCl_3$) and δ 77.2 ppm for ^{13}C ($CDCl_3$)]. Abbreviations used in the NMR follow-up experiments: *s*, singlet; *d*, doublet; *t*, triplet and *m*, multiplet. The pyrrole **1**, maleimides **2**, ChCl, and $ZnCl_2 \cdot 3H_2O$ were obtained from commercial sources and used as received.

Preparation of deep eutectic solvent (ChCl:$ZnCl_2$)

1.24 mmol of choline chloride and 2.5 mmol of $ZnCl_2$ were mixed in a 5 mL round-bottomed flask and heated for 15 minutes at 120°C until a clear liquid appeared. The mixture was allowed to stir for 15 min more, the colorless liquid was used directly for the reactions without purification.

Typical procedure for the pyrrole-maleimide Michael addition in ChCl:$ZnCl_2$

0.1 ml of water were added to the ChCl:$ZnCl_2$ (0.650 g) with stirring for 5 min, and then, the maleimide **2a** (0.072g, 0.74 mmol, 1 equiv) and pyrrole **1** (0.05 g, 0.74 mmol, 1 equiv)

were slowly added to the reaction mixture. The reaction mixture was stirred at room temperature for 2.5h. TLC monitored the reaction. After completion of the reaction, water was added, extraction was made with AcOEt, the organic phase was separated and dried on anhydrous Na_2SO4, and the solvent was evaporated. The pure **3a** product was purified on a silica gel chromatographic column using the mixture of Hex/EtOAc (7:3, v/v) as the eluent.

3-(1H-pyrrol-2-yl)pyrrolidine-2,5-dione) 3a

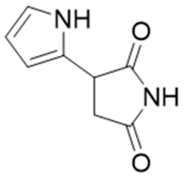

ACCORDING TO GENERAL PROCEDURE, 3A WAS OBTAINED IN 82% YIELD (0.102G). BROWN CRYSTALS, RF 0.28 (HEX/ETOAC 6:4), MELTING POINT (MEASURED): 105-108°C;
^{1}H NMR (400 MHz, CD_3OD; **ME$_4$SI**) δ_H = 10.32 (s, 1H), 6.71 (td, *J* = 2.7, 1.4 Hz, 3H), 6.04 (dd, *J* = 5.5, 2.8 Hz, 1H), 5.99 (dd, *J* = 2.4, 1.7 Hz, 1H), 4.15 (dd, *J* = 9.5, 5.3 Hz, 1H), 3.14 (dd, *J* = 18.2, 9.5 Hz, 1H), 2.82 (dd, *J* = 18.2, 5.3 Hz, 1H); **^{13}C {^{1}H} NMR (100 MHz, CD_3OD; ME$_4$SI)** δ_C **=** 179.67, 178.62, 126.53, 118.22, 107.54, 105.49, 40.94, 36.72; IR (v/cm^{-1}) 3352, 1769, 1682, 1188.

1-phenyl-3-(1H-pyrrol-2-yl)pyrrolidine-2,5-dione 3b

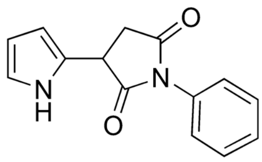

According to general procedure, **3b** was obtained in 87% yield (0.155g). Colorless crystals, rf 0.35 (Hex/EtOAc 7:3), melting point (measured): 178-180°C; 1H NMR (400 MHz, $CDCl_3$, Me4Si) δH = 9.13 (s, 1H), 7.43– 7.41 (m, 2H), 7.40-7.39 (ddd, J = 7.4, 3.7, 1.2

Hz, 1H), 7.29 – 7.26 (m, 2H), 6.83 (s, 1H), 6.22-6.20 (dd, J = 6.1, 2.8 Hz, 1H), 6.10 – 6.08 (m, 1H), 4.27- 4.24 (dd, J = 9.3, 5.6 Hz, 1H), 3.40 - 3.33 (dd, J = 18.3, 9.3 Hz, 1H), 3.25 - 3.19 (dd, J = 18.3, 5.6 Hz, 1H); 13C{1H} NMR (100 MHz, $CDCl_3$, Me4Si) δC = 176.94, 174.97, 131.78, 129.48, 129.08, 126.65, 125.41, 119.23, 108.71, 105.98, 38.71, 34.42; IR (v/cm-1) 3318, 2940, 1685, 1396

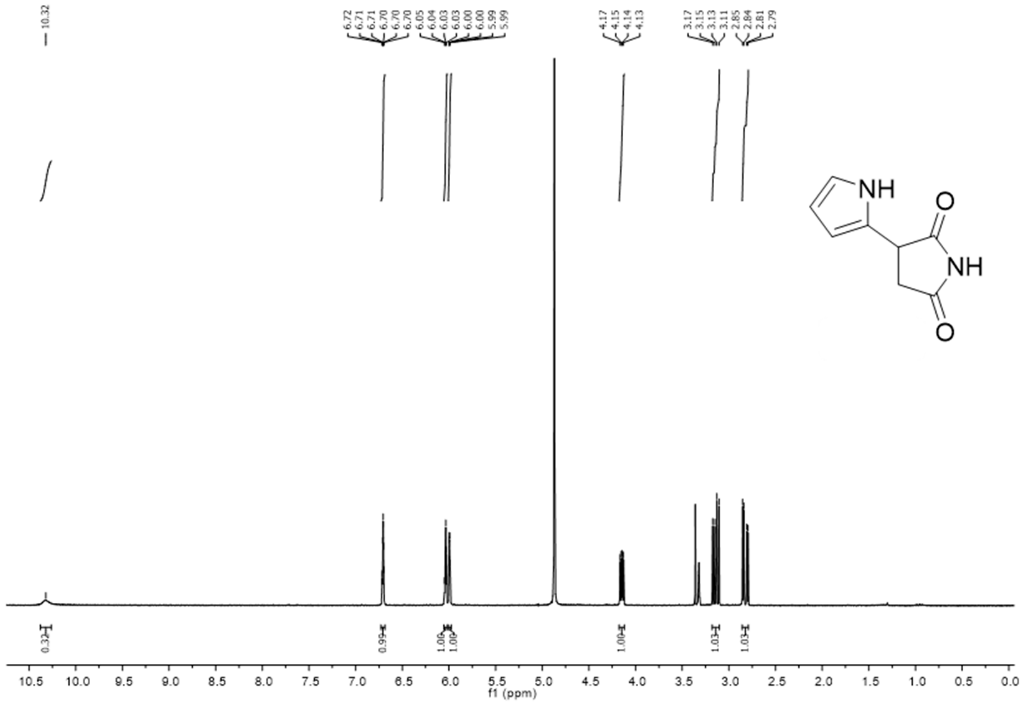

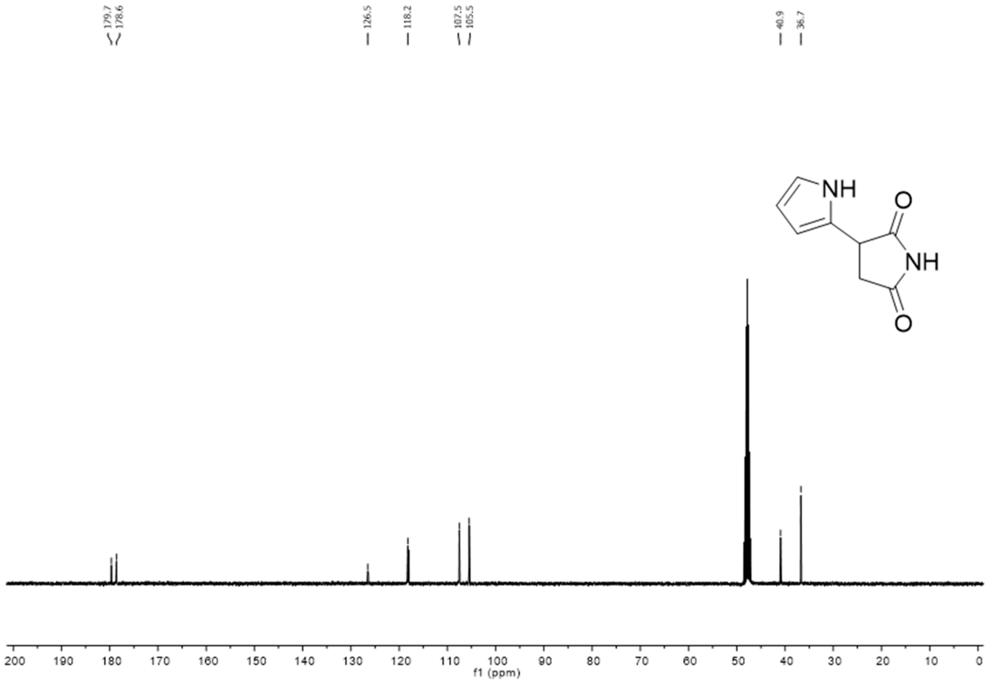

Figure S1. ^{1}H NMR (400MHz, CD_3OD), $^{13}C\{^{1}H\}$ NMR (100MHz, CD_3OD) *3-(1H-pyrrol-2-yl)pyrrolidine-2,5-dione)* ***3*****a**

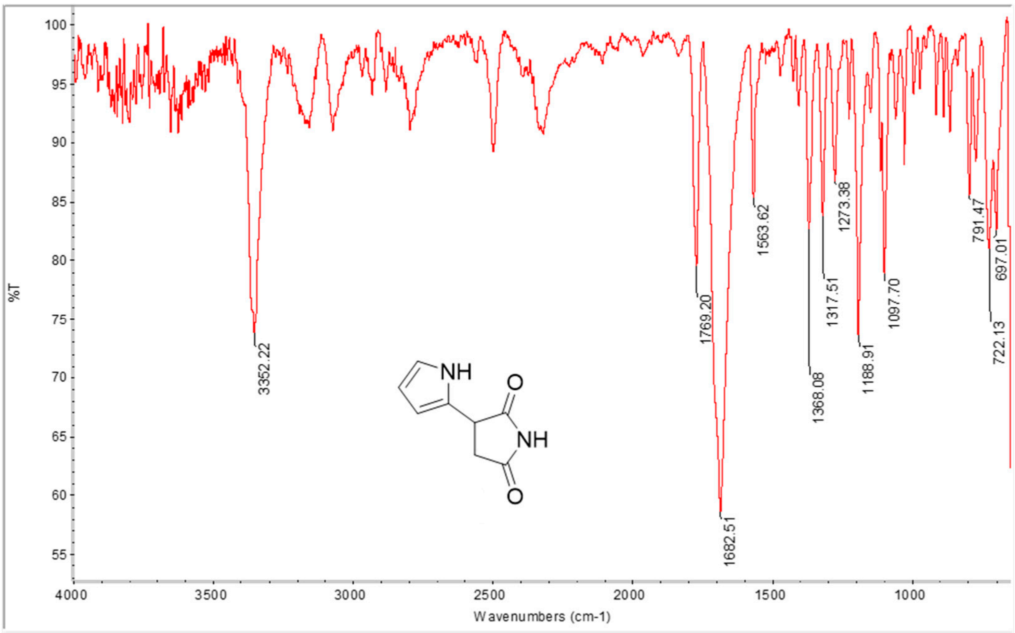

Figure S2. IR Spectrum 3-(1H-pyrrol-2-yl)pyrrolidine-2,5-dione ***3*****a**

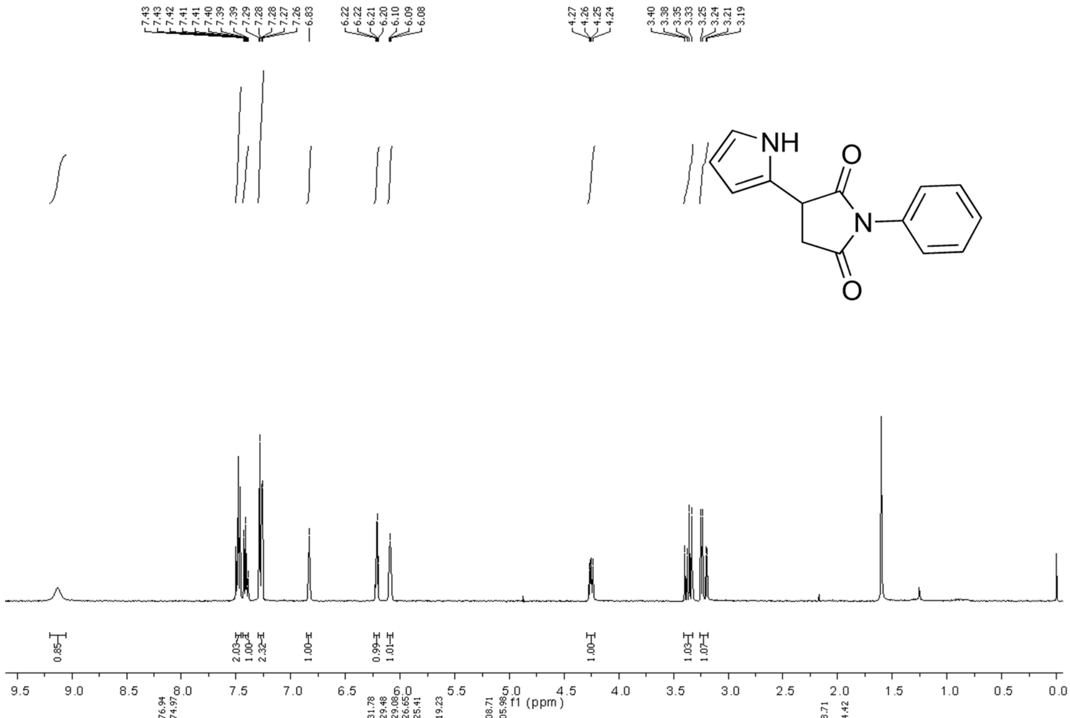

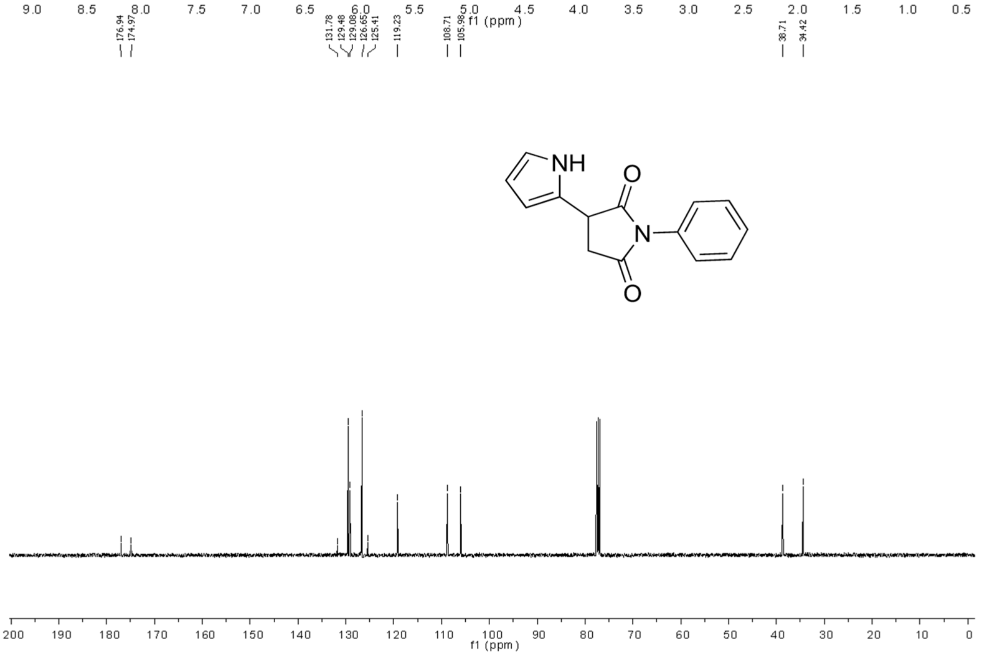

Figure S3 ^{1}H NMR (400MHz, $CDCl_3$), $^{13}C\{^{1}H\}$ NMR (100MHz, $CDCl_3$) **1-phenyl-3-(1*H*-pyrrol-2-yl)pyrrolidine-2,5-dione *3b***

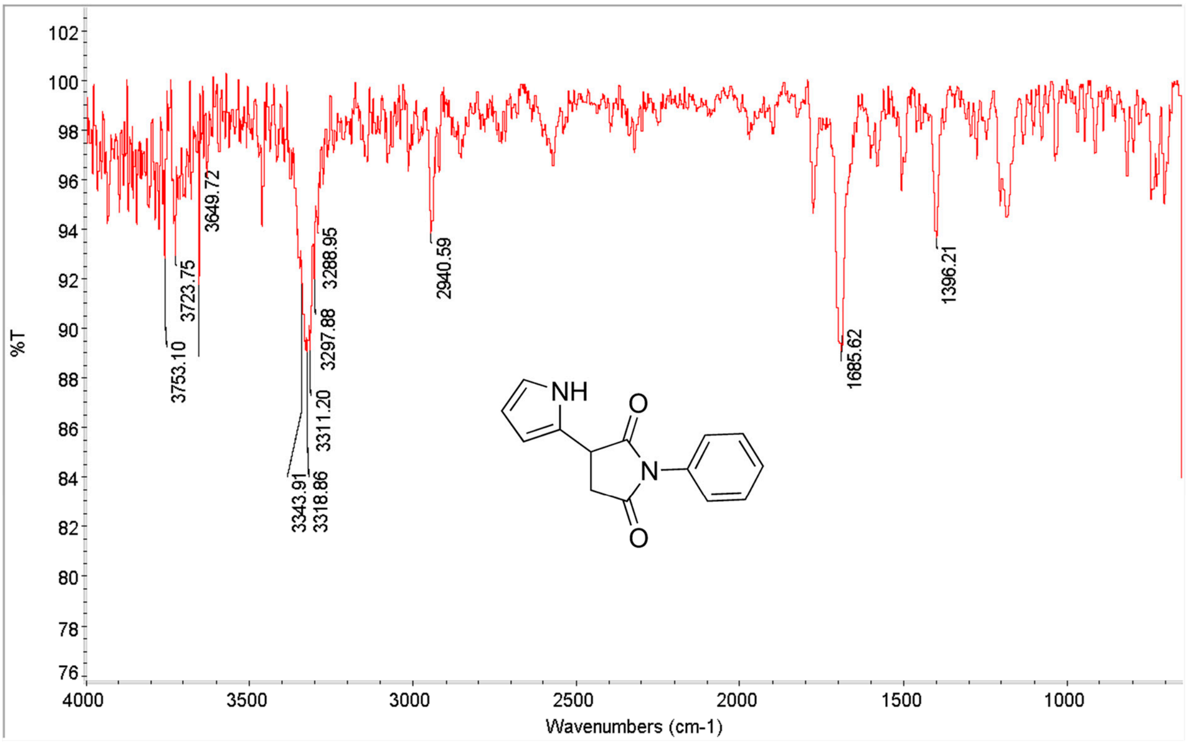

Figure S4. IR Spectrum1-PHENYL-3-(1H-PYRROL-2-YL)PYRROLIDINE-2,5-DIONE 3B

Reference Gaussian 09

Gaussian 09, Revision B.01,
M. J. Frisch, G. W. Trucks, H. B. Schlegel, G. E. Scuseria, M. A. Robb, J. R. Cheeseman, G. Scalmani, V. Barone, B. Mennucci, G. A. Petersson, H. Nakatsuji, M. Caricato, X. Li, H. P. Hratchian, A. F. Izmaylov, J. Bloino, G. Zheng, J. L. Sonnenberg, M. Hada, M. Ehara, K. Toyota, R. Fukuda, J. Hasegawa, M. Ishida, T. Nakajima, Y. Honda, O. Kitao, H. Nakai, T. Vreven, J. A. Montgomery, Jr., J. E. Peralta, F. Ogliaro, M. Bearpark, J. J. Heyd, E. Brothers, K. N. Kudin, V. N. Staroverov, T. Keith, R. Kobayashi, J. Normand, K. Raghavachari, A. Rendell, J. C. Burant, S. S. Iyengar, J. Tomasi, M. Cossi, N. Rega, J. M. Millam, M. Klene, J. E. Knox, J. B. Cross, V. Bakken, C. Adamo, J. Jaramillo, R. Gomperts, R. E. Stratmann, O. Yazyev, A. J. Austin, R. Cammi, C. Pomelli, J. W. Ochterski, R. L. Martin, K. Morokuma, V. G. Zakrzewski, G. A. Voth, P. Salvador, J. J. Dannenberg, S. Dapprich, A. D. Daniels, O. Farkas, J. B. Foresman, J. V. Ortiz, J. Cioslowski, and D. J. Fox, Gaussian, Inc., Wallingford CT, 2010.

Theoretical calculations

Energy and coordinates of Pirrol

	(Hartree/Particle)
Zero-point correction=	0.076524
Thermal correction to Energy=	0.080526
Thermal correction to Enthalpy=	0.081470
Thermal correction to Gibbs Free Energy=	0.050727
Sum of electronic and zero-point Energies=	0.117711
Sum of electronic and thermal Energies=	0.121713
Sum of electronic and thermal Enthalpies=	0.122657
Sum of electronic and thermal Free Energies=	0.091914

	E (Thermal)	CV	S
	KCal/Mol	Cal/Mol-Kelvin	Cal/Mol-Kelvin
Total	50.531	14.763	64.705

Charge:0 Multiplicity: 1

C	0.00000000	1.13894600	0.33389500
C	0.00000000	0.71289600	-0.99413900
C	0.00000000	-0.71289600	-0.99413900
C	0.00000000	-1.13894600	0.33389500
N	0.00000000	0.00000000	1.14807800
H	0.00000000	2.13405300	0.73293900
H	0.00000000	1.33799600	-1.86318700
H	0.00000000	-1.33799600	-1.86318700
H	0.00000000	-2.13405300	0.73293900
H	0.00000000	0.00000000	2.14687600

Energy and coordinates of Maleimide

	(Hartree/Particle)
Zero-point correction=	0.063680
Thermal correction to Energy=	0.069254
Thermal correction to Enthalpy=	0.070198
Thermal correction to Gibbs Free Energy=	0.034300
Sum of electronic and zero-point Energies=	-0.018407

Sum of electronic and thermal Energies= -0.012834
Sum of electronic and thermal Enthalpies= -0.011890
Sum of electronic and thermal Free Energies= -0.047787

	E (Thermal)	CV	S
	KCal/Mol	Cal/Mol-Kelvin	Cal/Mol-Kelvin
Total	43.457	20.306	75.553

Charge:0 Multiplicity: 1

C	0.00055600	1.28265100	0.66880100
C	0.00055600	1.28265100	-0.66880100
C	-0.00017900	-0.14554100	-1.18416400
N	-0.00048800	-0.95913900	0.00000000
C	-0.00017900	-0.14554100	1.18416400
O	-0.00017900	-0.57343900	2.30441300
O	-0.00017900	-0.57343900	-2.30441300
H	0.00098500	2.10874300	1.36391100
H	0.00098500	2.10874300	-1.36391100
H	-0.00020500	-1.97379100	0.00000000

Energy and coordinates of N-Phenyl Maleimide

	(Hartree/Particle)
Zero-point correction=	0.140030
Thermal correction to Energy=	0.150379
Thermal correction to Enthalpy=	0.151323
Thermal correction to Gibbs Free Energy=	0.103037
Sum of electronic and zero-point Energies=	0.105272
Sum of electronic and thermal Energies=	0.115621
Sum of electronic and thermal Enthalpies=	0.116565
Sum of electronic and thermal Free Energies=	0.068279

	E (Thermal)	CV	S
	KCal/Mol	Cal/Mol-Kelvin	Cal/Mol-Kelvin
Total	94.364	39.805	101.627

Charge:0 Multiplicity: 1

C	-3.02068500	-0.66403500	-0.06940200

C	-3.02068600	0.66403200	0.06942000
C	-1.59625700	1.17962300	0.11953500
N	-0.75995200	0.00000100	-0.00000800
C	-1.59625600	-1.17962100	-0.11955700
O	-1.21205000	-2.31000100	-0.22953700
O	-1.21205300	2.31000100	0.22953600
C	0.68360600	0.00000100	-0.00000300
C	1.38183600	-1.19314500	0.25828500
C	2.77785200	-1.18193100	0.25372000
C	3.48047700	-0.00000100	0.00000500
C	2.77785500	1.18193000	-0.25371500
C	1.38183900	1.19314600	-0.25828700
H	-3.84645700	-1.35696500	-0.14265400
H	-3.84645800	1.35695900	0.14269400
H	0.84921200	-2.12708800	0.45123900
H	3.32064600	-2.10654000	0.45096800
H	4.56820200	-0.00000200	0.00000800
H	3.32065200	2.10653800	-0.45095900
H	0.84921800	2.12709000	-0.45124500

Energy and coordinates of Choline chloride

	(Hartree/Particle)
Zero-point correction=	0.174838
Thermal correction to Energy=	0.185135
Thermal correction to Enthalpy=	0.186079
Thermal correction to Gibbs Free Energy=	0.139259
Sum of electronic and zero-point Energies=	0.058852
Sum of electronic and thermal Energies=	0.069149
Sum of electronic and thermal Enthalpies=	0.070093
Sum of electronic and thermal Free Energies=	0.023273

	E (Thermal) KCal/Mol	CV Cal/Mol-Kelvin	S Cal/Mol-Kelvin
Total	116.174	38.440	98.540

Charge:0 Multiplicity: 1

C	2.01643700	0.78994400	-0.80643600
C	0.44319800	0.37871400	1.09187000
H	2.59453400	1.47187500	-0.16612500
H	2.68736100	0.34525900	-1.55552400
C	-0.38477200	1.60709500	0.57877100
H	-0.28828100	-0.38079500	1.47039200
H	1.07965900	0.68573600	1.94161500
H	-1.17932300	1.80527000	1.32867000
H	0.25888100	2.49911800	0.48002000
N	1.29292800	-0.26498200	0.00136900
C	0.43589000	-1.08555800	-0.93224200
H	-0.33380500	-0.41247100	-1.41589300
H	1.01856000	-1.57747100	-1.71777300
H	-0.17010500	-1.83920000	-0.39141900
C	2.31407600	-1.17546200	0.65516800
H	2.92725700	-1.70120400	-0.09618300
H	2.99678800	-0.61474000	1.31437000
H	1.81472400	-1.94930200	1.26777600
H	1.26167300	1.40912900	-1.35892100
O	-0.93161400	1.34684500	-0.66348300
Cl	-2.55180800	-0.72247700	0.06889700
H	-1.83375600	0.53261700	-0.49675400

Energy and coordinates of Zinc chloride

	(Hartree/Particle)
Zero-point correction=	0.002017
Thermal correction to Energy=	0.006608
Thermal correction to Enthalpy=	0.007552
Thermal correction to Gibbs Free Energy=	-0.024785
Sum of electronic and zero-point Energies=	-0.080082
Sum of electronic and thermal Energies=	-0.075491
Sum of electronic and thermal Enthalpies=	-0.074547
Sum of electronic and thermal Free Energies=	-0.106884

	E (Thermal)	CV	S
	KCal/Mol	Cal/Mol-Kelvin	Cal/Mol-Kelvin
Total	4.146	12.035	68.059

Charge:0 Multiplicity: 1

Zn	0.00000000	0.00000000	0.00000700
Cl	0.00000000	0.00000000	-2.03593600
Cl	0.00000000	0.00000000	2.03592400

Energy and coordinates of Zinc zincate (2 $ZnCl_2$. 3 H_2O)

	(Hartree/Particle)
Zero-point correction=	0.118262
Thermal correction to Energy=	0.144957
Thermal correction to Enthalpy=	0.145902
Thermal correction to Gibbs Free Energy=	0.058447
Sum of electronic and zero-point Energies=	-0.783931
Sum of electronic and thermal Energies=	-0.757236
Sum of electronic and thermal Enthalpies=	-0.756292
Sum of electronic and thermal Free Energies=	-0.843747

	E (Thermal)	CV	S
	KCal/Mol	Cal/Mol-Kelvin	Cal/Mol-Kelvin
Total	90.962	84.132	184.065

Charge:0 Multiplicity: 1

Zn	-0.32830500	-0.10237900	-0.46168000
Zn	1.45309700	-0.09869300	-0.01696400
Cl	0.08996000	0.73640500	1.92090200
Cl	0.33653600	-2.38088800	-0.43790100
Cl	0.81327300	1.61928200	-1.73851600
Cl	3.58183900	-0.27087200	0.37265600
O	-0.80386100	3.06734100	0.27530500
H	-0.63393400	3.48553300	-0.57084100
O	-1.62344800	0.04118000	-2.02399300
H	-2.39951700	-0.65790900	-2.09953300
O	-1.31292000	-1.77232900	2.61069500
H	-0.51504100	-2.17825700	2.24118300
O	-2.15544400	-0.44320200	0.50620700

H	-2.61100000	0.46927000	0.80154700
O	-3.12079100	-1.84813500	-1.41404400
H	-3.29622500	-1.43303400	-0.52811600
O	-2.82298100	1.94792100	1.18407700
H	-2.06198000	2.20005100	1.74615300
H	-2.66440700	2.44005000	0.35198100
H	0.02951100	3.07903000	0.75836400
H	-1.00641200	-1.16354500	3.29641700
H	-2.04127500	-0.93651600	1.40911600
H	-2.42273000	-2.50346700	-1.24176000
H	-1.37249200	0.32199900	-2.90254100

Structures for the proposed reactions between Pirrol et Maleimide

Transition state a) TS1

	(Hartree/Particle)
Zero-point correction=	0.141535
Thermal correction to Energy=	0.151409
Thermal correction to Enthalpy=	0.152353
Thermal correction to Gibbs Free Energy=	0.105257
Sum of electronic and zero-point Energies=	0.137864
Sum of electronic and thermal Energies=	0.147738
Sum of electronic and thermal Enthalpies=	0.148682
Sum of electronic and thermal Free Energies=	0.101586

	E (Thermal)	CV	S
	KCal/Mol	Cal/Mol-Kelvin	Cal/Mol-Kelvin
Total	95.011	37.667	99.123

Charge:0 Multiplicity: 1

C	2.30579600	-0.75452800	0.00453000
C	1.06778000	-1.08094500	0.65096200
C	0.20670200	0.03655200	0.71721000
C	1.03411700	1.24687300	0.19948500
N	2.24719700	0.71289000	-0.24331700

O	0.68229100	2.40558300	0.14581100
O	3.28955700	-1.36329200	-0.33906500
N	-1.72892600	-1.32707400	-0.31281800
C	-2.98485600	-1.05563600	0.11335800
C	-3.19198900	0.36456000	0.07503200
H	-3.70463100	-1.80640400	0.41938200
C	-2.02474400	0.95682300	-0.36716500
H	-4.11259400	0.85284300	0.34515400
H	-1.26726300	-2.22788400	-0.35076300
H	-1.81413100	2.01114900	-0.50096400
C	-1.01435800	-0.08473400	-0.56824000
H	-0.38374800	-0.07350400	-1.47394500
H	-0.41884600	0.21716500	1.59749900
H	0.89938500	-2.04214500	1.08042400
H	3.00846700	1.25595000	-0.62885000

Intermediate a) Int1

	(Hartree/Particle)
Zero-point correction=	0.142427
Thermal correction to Energy=	0.152525
Thermal correction to Enthalpy=	0.153470
Thermal correction to Gibbs Free Energy=	0.105524
Sum of electronic and zero-point Energies=	0.135961
Sum of electronic and thermal Energies=	0.146059
Sum of electronic and thermal Enthalpies=	0.147004
Sum of electronic and thermal Free Energies=	0.099058

	E (Thermal)	CV	S
	KCal/Mol	Cal/Mol-Kelvin	Cal/Mol-Kelvin
Total	95.711	38.517	100.911

Charge:0 Multiplicity: 1

C	2.01653200	-0.98445500	0.15115900
C	0.73519300	-0.93374800	0.72458400
C	0.16392000	0.43947200	0.66719100
C	1.29358500	1.28606100	0.00797500
N	2.32740100	0.41393300	-0.29708500
O	1.22947100	2.47376200	-0.24849600
O	2.84204300	-1.84076200	-0.08946200
N	-1.39740000	-0.98643000	-0.60342300
C	-2.66373900	-1.23778000	-0.31461000
C	-3.30953200	-0.01086600	0.17553000
H	-3.15910100	-2.20498700	-0.41015400
C	-2.37653200	0.97366000	0.22755800
H	-4.35046000	0.03191000	0.45658400
H	-0.58133900	-1.64096400	-0.74537300
H	-2.48418600	2.00056400	0.55152200
C	-1.06103700	0.45042200	-0.29801100
H	-0.79148900	0.98430500	-1.25619700
H	-0.11166400	0.85548300	1.65611200
H	0.39614300	-1.67185700	1.41182600
H	3.20963200	0.69243000	-0.70534300

Transition state a)TS2

	(Hartree/Particle)
Zero-point correction=	0.140125
Thermal correction to Energy=	0.149829
Thermal correction to Enthalpy=	0.150773
Thermal correction to Gibbs Free Energy=	0.104239
Sum of electronic and zero-point Energies=	0.156379
Sum of electronic and thermal Energies=	0.166082
Sum of electronic and thermal Enthalpies=	0.167027
Sum of electronic and thermal Free Energies=	0.120492

	E (Thermal)	CV	S
	KCal/Mol	Cal/Mol-Kelvin	Cal/Mol-Kelvin
Total	94.019	37.556	97.940

Charge:0 Multiplicity: 1

C	2.30807600	-0.72951900	-0.23211500
C	1.14684100	-1.23661200	0.38894100
C	0.25844400	-0.11727500	0.87094900
C	1.01276500	1.17092400	0.44368200
N	2.18035700	0.76304800	-0.18935700
O	0.62666500	2.31374900	0.59320800
O	3.28044200	-1.18714000	-0.79086200
N	-2.10371000	-1.06567400	0.57541300
C	-3.25984900	-0.62166100	0.04049700
C	-2.97410800	0.46277400	-0.86099400
H	-4.24206200	-1.02782200	0.26655600
C	-1.61182100	0.67560900	-0.86016000
H	-3.72402200	1.01174800	-1.40707500
H	-1.97704200	-1.80717800	1.25215700
H	-1.05193700	1.43380100	-1.39591400
C	-0.97499300	-0.33923100	-0.01214200
H	-0.26085000	-1.17008000	-0.70788700
H	0.03965600	-0.09814500	1.95295700
H	1.10170500	-2.22309600	0.78365100
H	2.88903500	1.39623400	-0.53756200

Succinimide a)P

	(Hartree/Particle)
Zero-point correction=	0.144407
Thermal correction to Energy=	0.154517
Thermal correction to Enthalpy=	0.155461
Thermal correction to Gibbs Free Energy=	0.106484
Sum of electronic and zero-point Energies=	0.045782
Sum of electronic and thermal Energies=	0.055892
Sum of electronic and thermal Enthalpies=	0.056836
Sum of electronic and thermal Free Energies=	0.007859

	E (Thermal)	CV	S
	KCal/Mol	Cal/Mol-Kelvin	Cal/Mol-Kelvin
Total	96.961	38.204	103.081

Charge:0 Multiplicity: 1

C	2.30466600	-0.73315000	-0.11221000
C	1.09546100	-1.15028600	0.70118600
C	0.22090000	0.11385200	0.88045700
C	0.99186200	1.24357100	0.17159800
N	2.17198000	0.66353800	-0.38665900
O	0.72662400	2.41066200	0.06809800
O	3.23541900	-1.39164400	-0.49624300
N	-1.45495500	-0.09568000	-1.02333700
C	-2.83154400	-0.29105100	-1.17370600
C	-3.39429500	-0.38948900	0.09923400
H	-3.30441100	-0.34482100	-2.13681000
C	-2.34805200	-0.24268400	1.05339800
H	-4.43004900	-0.54561700	0.32686300
H	-0.79903800	0.04097900	-1.76757500
H	-2.46476400	-0.26671800	2.11784100
C	-1.15471900	-0.05848100	0.34871600
H	0.54598200	-1.97093100	0.19486600
H	0.15107500	0.38281900	1.96908600
H	1.40847100	-1.57791400	1.67366700
H	2.87154000	1.20136100	-0.89485600

Transition state b)TS1

	(Hartree/Particle)
Zero-point correction=	0.144186
Thermal correction to Energy=	.160252
Thermal correction to Enthalpy=	0.161196
Thermal correction to Gibbs Free Energy=	0.094857

Sum of electronic and zero-point Energies= 0.022698
Sum of electronic and thermal Energies= 0.038764
Sum of electronic and thermal Enthalpies= 0.039708
Sum of electronic and thermal Free Energies= -0.026631

	E (Thermal)	CV	S
	KCal/Mol	Cal/Mol-Kelvin	Cal/Mol-Kelvin
Total	100.559	54.409	139.622

Charge:0 Multiplicity: 1

C	-0.01824300	-0.17996400	0.58906800
C	-1.22862300	-0.83386300	1.01108400
C	-2.30733700	0.05355000	0.95132800
C	-1.71566800	1.44503100	0.60819300
N	-0.33362200	1.21085400	0.36287900
O	-2.27500500	2.50629900	0.52666700
O	1.10447100	-0.66435600	0.43755600
N	-3.67445800	-1.61455900	-0.58234800
C	-5.01356700	-1.64328300	-0.34789300
C	-5.51152500	-0.30291700	-0.36685900
H	-5.58473900	-2.55010200	-0.18756300
C	-4.44209200	0.54760100	-0.60610600
H	-6.54378900	-0.02991000	-0.22896400
H	-3.03734300	-2.39937200	-0.59722400
Zn	2.92962700	-0.10108100	-0.11507000
Cl	2.81390400	1.99605100	-0.57705100
Cl	4.03472800	-1.91614400	0.00636900
H	-4.45664500	1.62729900	-0.67666600
C	-3.22795100	-0.24580100	-0.67780000
H	-2.41850100	-0.02377400	-1.38069500
H	-3.16582400	0.00747000	1.62223300
H	-1.23619900	-1.84851300	1.35507400
H	0.33835700	1.93919300	0.10392300

Intermediate b)Int1

	(Hartree/Particle)
Zero-point correction=	0.146002
Thermal correction to Energy=	0.162094
Thermal correction to Enthalpy=	0.163038
Thermal correction to Gibbs Free Energy=	0.095537
Sum of electronic and zero-point Energies=	0.012417
Sum of electronic and thermal Energies=	0.028509
Sum of electronic and thermal Enthalpies=	0.029453
Sum of electronic and thermal Free Energies=	-0.038048

	E (Thermal)	CV	S
	KCal/Mol	Cal/Mol-Kelvin	Cal/Mol-Kelvin
Total	101.715	54.735	142.067

Charge:0 Multiplicity: 1

C	-0.05903200	-0.13479900	0.54020000
C	-1.27151400	-0.73474600	0.84997600
C	-2.37475100	0.25575900	0.77963200
C	-1.64925300	1.59876100	0.44735500
N	-0.29622400	1.29182800	0.28526800
O	-2.18536800	2.67391500	0.29965400
O	1.08050600	-0.63037900	0.44584200
N	-3.52083200	-1.54984600	-0.51327400
C	-4.81418800	-1.84482500	-0.44924900
C	-5.59094900	-0.60974000	-0.32373100
H	-5.23947100	-2.85109200	-0.48475600
C	-4.73109700	0.44238400	-0.29254000
H	-6.67040600	-0.59601300	-0.25948000
H	-2.70263500	-2.17368100	-0.53194800
Zn	2.87580900	-0.15568100	-0.06874500
Cl	2.92911900	1.95008800	-0.57624200
Cl	4.03406400	-1.95796300	0.02763700
H	-4.94045900	1.50409200	-0.19558800
C	-3.31411700	-0.06228100	-0.41955100
H	-2.83197200	0.30002200	-1.37614100
H	-2.95091700	0.34326800	1.72439400

H	-1.35783900	-1.70651000	1.27567000
H	0.43300100	1.97899500	0.07602900

Transition state b)TS2

	(Hartree/Particle)
Zero-point correction=	0.142921
Thermal correction to Energy=	0.158685
Thermal correction to Enthalpy=	0.159629
Thermal correction to Gibbs Free Energy=	0.094251
Sum of electronic and zero-point Energies=	0.035552
Sum of electronic and thermal Energies=	0.051315
Sum of electronic and thermal Enthalpies=	0.052259
Sum of electronic and thermal Free Energies=	-0.013119

	E (Thermal)	CV	S
	KCal/Mol	Cal/Mol-Kelvin	Cal/Mol-Kelvin
Total	99.576	54.059	137.600

Charge:0 Multiplicity: 1

C	0.01677500	-0.37886300	-0.67144100
C	1.18434300	-1.12800100	-0.90692600
C	2.34787300	-0.18896300	-1.15853600
C	1.71742300	1.22323300	-1.04769500
N	0.35296600	1.03306000	-0.74066000
O	2.27070700	2.28877100	-1.16213900
O	-1.12806400	-0.77322800	-0.40041700
N	4.37920800	-1.36121600	-0.11712100
C	5.30961300	-1.05497900	0.81438100
C	4.84086400	0.05543000	1.59658000
H	6.25554100	-1.57716000	0.93563500
C	3.60095800	0.42631100	1.11723900
H	5.39780400	0.51371400	2.39917500
H	4.42641900	-2.08490000	-0.82248200

Zn	-2.85178500	-0.06615400	0.18973400
Cl	-2.54887900	2.02051300	0.66674900
Cl	-4.21690300	-1.70905700	0.12782900
H	2.97256900	1.24067300	1.45828700
C	3.20837500	-0.50952600	0.06157100
H	2.17602400	-1.24707300	0.48348300
H	2.86480100	-0.30873300	-2.12850900
H	1.16833400	-2.14231700	-1.24155300
H	-0.31335300	1.80053800	-0.61000200

Succinimide + $ZnCl_2$ b) P

	(Hartree/Particle)
Zero-point correction=	0.147164
Thermal correction to Energy=	0.163233
Thermal correction to Enthalpy=	0.164177
Thermal correction to Gibbs Free Energy=	0.097840
Sum of electronic and zero-point Energies=	-0.062618
Sum of electronic and thermal Energies=	-0.046550
Sum of electronic and thermal Enthalpies=	-0.045606
Sum of electronic and thermal Free Energies=	-0.111942

	E (Thermal)	CV	S
	KCal/Mol	Cal/Mol-Kelvin	Cal/Mol-Kelvin
Total	102.430	54.678	139.617

Charge:0 Multiplicity: 1

C	0.04939100	-0.24934000	0.43772500
C	-1.17164900	-1.07082000	0.79198300
C	-2.32418700	-0.05712700	0.97724200
C	-1.72558500	1.30035700	0.56589600
N	-0.32350100	1.09921500	0.31626100
O	-2.26042200	2.37069600	0.46371500
O	1.17447500	-0.70983700	0.28662200
N	-4.45228300	0.57947700	-0.24731400

C	-5.51102800	-0.05919500	-0.89569200
C	-5.28084400	-1.43553300	-0.84739900
H	-6.33024700	0.48267400	-1.33305400
C	-4.05440600	-1.65014400	-0.15952700
H	-5.91267900	-2.20099800	-1.25329800
H	-4.33564300	1.57529000	-0.13338300
Zn	3.04973100	-0.05088900	-0.17228500
Cl	2.80835600	2.04764300	-0.47427200
Cl	4.06731100	-1.89643300	-0.04734200
H	-3.61590400	-2.60524700	0.04561700
C	-3.54787900	-0.39643500	0.20084900
H	-1.38405300	-1.80724500	-0.01852200
H	-2.60176600	0.00734700	2.06714000
H	-0.99798700	-1.68788800	1.69741700
H	0.31517800	1.86388800	0.05229000

Transition state c)TS1

	(Hartree/Particle)
Zero-point correction=	0.317936
Thermal correction to Energy=	0.346915
Thermal correction to Enthalpy=	0.347859
Thermal correction to Gibbs Free Energy=	0.253110
Sum of electronic and zero-point Energies=	0.037005
Sum of electronic and thermal Energies=	0.065984
Sum of electronic and thermal Enthalpies=	0.066928
Sum of electronic and thermal Free Energies=	-0.027822

	E (Thermal)	CV	S
	KCal/Mol	Cal/Mol-Kelvin	Cal/Mol-Kelvin
Total	217.693	100.118	199.417

Charge:0 Multiplicity: 1

C	-0.59614100	1.20968800	0.52763900

C	-1.55989200	0.26595100	1.05815800
C	-2.83588900	0.76868000	0.92324800
C	-2.71884000	2.20032200	0.38308400
N	-1.29602400	2.37806000	0.13777800
O	-3.52833000	3.05707900	0.18063700
O	0.64014200	1.04588300	0.42239000
N	-3.73729000	-1.41064600	-0.49207300
C	-5.04733000	-1.64660800	-0.21385300
C	-5.80581000	-0.46522700	-0.48696200
H	-5.41639300	-2.59526000	0.14642400
C	-4.91515900	0.50400400	-0.93823500
H	-6.86681100	-0.37103400	-0.37094100
C	3.41729200	-2.17855000	1.17990900
C	1.54359200	-1.92754200	-0.46693600
H	3.61963200	-1.08920900	1.13975000
H	3.71507500	-2.53756200	2.17809600
C	2.46429100	-2.30250900	-1.62843000
H	0.49371000	-2.28163500	-0.68440500
H	1.45819900	-0.81005000	-0.40032300
H	2.14678400	-1.76629200	-2.54566100
H	3.53268100	-2.07163300	-1.44232700
N	1.96272100	-2.47800900	0.89213200
C	1.74694600	-3.97264100	0.93896000
H	2.29380700	-4.47398900	0.11069600
H	2.06822000	-4.40759900	1.89506700
H	0.66912100	-4.22609000	0.80032900
C	1.11823500	-1.81124700	1.95679500
H	1.37700700	-2.16145000	2.96642400
H	1.22748400	-0.70835500	1.92184800
H	0.03284400	-2.03069500	1.79858200
H	4.08171500	-2.66179600	0.43886200
O	2.46547000	-3.71301200	-1.86264400
H	-2.88384900	-2.11690200	-0.33190100
Cl	-1.54611800	-3.11952700	0.14973500

H	1.59624100	-4.00617500	-2.22321500
Zn	2.13942800	2.22248600	-0.14482000
Cl	1.56337500	4.27502800	0.04910500
Cl	3.63143000	0.77563600	-0.71151800
H	-5.14553600	1.51175600	-1.23432400
C	-3.59170000	-0.05597400	-0.89314600
H	-2.74620200	0.24843500	-1.48809200
H	-3.73028800	0.45104500	1.45142200
H	-1.29528500	-0.71880100	1.41697300
H	-0.88036200	3.24387200	-0.21247700

Intermediate c)Int1

	(Hartree/Particle)
Zero-point correction=	0.319984
Thermal correction to Energy=	0.348763
Thermal correction to Enthalpy=	0.349707
Thermal correction to Gibbs Free Energy=	0.256253
Sum of electronic and zero-point Energies=	-0.009101
Sum of electronic and thermal Energies=	0.019678
Sum of electronic and thermal Enthalpies=	0.020622
Sum of electronic and thermal Free Energies=	-0.072831

	E (Thermal)	CV	S
	KCal/Mol	Cal/Mol-Kelvin	Cal/Mol-Kelvin
Total	218.852	100.688	196.690

Charge:0 Multiplicity: 1

C	0.32181100	-1.18916600	0.42000900
C	1.37677100	-0.34544900	0.64544400
C	2.67153500	-1.09718500	0.56941300
C	2.22009200	-2.54704200	0.23958700
N	0.80935700	-2.53375300	0.18677200
O	2.91340500	-3.51995900	0.05071000

O	-0.92953900	-0.90348500	0.38792100
N	4.05427700	0.83670400	-0.25219700
C	5.36704100	0.85888100	-0.21464300
C	5.94211900	-0.48813200	-0.44020100
H	5.96360700	1.75187900	-0.04238000
C	4.91404200	-1.34327800	-0.62317800
H	6.99918400	-0.68675400	-0.44166700
C	-2.95623100	2.44172700	1.25660100
C	-1.17674600	2.01961400	-0.45730700
H	-3.22993500	1.36576800	1.32457500
H	-3.20693500	2.91211800	2.21954600
C	-2.06688900	2.52682500	-1.59110900
H	-0.09978000	2.20940200	-0.69101300
H	-1.26563700	0.88920600	-0.39426900
H	-1.91044000	1.91259000	-2.50030200
H	-3.14980000	2.51032700	-1.34436600
N	-1.49323000	2.60920400	0.91393000
C	-1.13619900	4.07768500	0.93935000
H	-1.64022000	4.61780700	0.10503700
H	-1.41855200	4.55561500	1.88837300
H	-0.04727500	4.22799300	0.79521900
C	-0.67429600	1.87265700	1.95109300
H	-0.83443500	2.26789100	2.96368500
H	-0.92389600	0.78443400	1.95185500
H	0.41537900	1.93053200	1.72355700
H	-3.60908200	2.88026800	0.47769400
O	-1.81421700	3.90726500	-1.87375800
H	3.13171100	1.93855200	-0.09517700
Cl	2.30019900	3.08578500	0.04167100
H	-0.95394600	4.01873600	-2.33417500
Zn	-2.51126400	-1.83005300	-0.10219600
Cl	-2.44167000	-3.97332600	0.05504900
Cl	-3.80019900	-0.17274600	-0.69069900
H	4.91653600	-2.41318500	-0.78916500

C	3.60216700	-0.56758400	-0.53415400
H	3.06410300	-0.56678600	-1.52140300
H	3.19674200	-1.09767600	1.55499600
H	1.34415300	0.69804100	0.85774200
H	0.22350900	-3.35325800	0.03117700

Transition state c)TS2

	(Hartree/Particle)
Zero-point correction=	0.316591
Thermal correction to Energy=	0.344927
Thermal correction to Enthalpy=	0.345872
Thermal correction to Gibbs Free Energy=	0.253404
Sum of electronic and zero-point Energies=	0.042206
Sum of electronic and thermal Energies=	0.070543
Sum of electronic and thermal Enthalpies=	0.071487
Sum of electronic and thermal Free Energies=	-0.020980

	E (Thermal)	CV	S
	KCal/Mol	Cal/Mol-Kelvin	Cal/Mol-Kelvin
Total	216.445	99.341	194.615

Charge:0 Multiplicity: 1

C	-0.10326100	-1.27471200	-0.53695700
C	-1.24829300	-0.47081100	-0.69853600
C	-2.46380700	-1.36038500	-0.95604200
C	-1.89075300	-2.78549500	-0.83982300
N	-0.47274700	-2.64480000	-0.59217200
O	-2.42246400	-3.85798400	-0.92430300
O	1.06745000	-0.85643000	-0.32365200
N	-4.12794400	0.23397900	0.16153000
C	-5.10946100	0.12160000	1.07249400

C	-5.04057500	-1.17117100	1.71575100
H	-5.83180100	0.90150200	1.27834500
C	-3.95416000	-1.83699300	1.18951300
H	-5.73290900	-1.52036200	2.45738600
C	2.51938900	3.07340100	-1.03446500
C	0.67898600	2.18641900	0.41348600
H	3.04555200	2.09801500	-0.95958400
H	2.78474800	3.52247000	-2.00497300
C	1.30177500	2.79838400	1.66984700
H	-0.43892600	2.15547600	0.51108100
H	1.01063200	1.10780700	0.33596900
H	1.08911700	2.15297000	2.54593300
H	2.40122400	2.93003100	1.59666500
N	1.02425300	2.90102400	-0.88984600
C	0.35592000	4.25497900	-0.93805300
H	0.63517100	4.86404100	-0.04960000
H	0.61964300	4.81432300	-1.84686000
H	-0.75347900	4.15717400	-0.91617300
C	0.52362200	2.05472700	-2.04081100
H	0.68620700	2.54253200	-3.01343400
H	1.02055100	1.06329600	-2.05453600
H	-0.57551700	1.86246500	-1.94571000
H	2.92961000	3.72423200	-0.23959800
O	0.82176600	4.12360600	1.90677100
H	-3.71380300	1.24559300	-0.34252300
Cl	-2.79676200	2.55690700	-0.61594000
H	-0.11523600	4.10790000	2.20772800
Zn	2.76943400	-1.56722900	0.25248700
Cl	2.88131300	-3.68938800	0.01202700
Cl	3.63575300	0.31091200	0.88072000
H	-3.61414700	-2.83212100	1.42107800
C	-3.30053900	-0.95135400	0.24179400
H	-2.04302000	-0.34808200	0.62909900
H	-2.97109100	-1.21410300	-1.93046900

H	-1.24214400	0.54499700	-1.05086300
H	0.16940600	-3.43313100	-0.48557600

Succinimide + ChCl + ZnCl2 : c)P

	(Hartree/Particle)
Zero-point correction=	0.323057
Thermal correction to Energy=	0.352081
Thermal correction to Enthalpy=	0.353025
Thermal correction to Gibbs Free Energy=	0.256845
Sum of electronic and zero-point Energies=	-0.033326
Sum of electronic and thermal Energies=	-0.004303
Sum of electronic and thermal Enthalpies=	-0.003359
Sum of electronic and thermal Free Energies=	-0.099538

	E (Thermal)	CV	S
	KCal/Mol	Cal/Mol-Kelvin	Cal/Mol-Kelvin
Total	220.934	100.048	202.427

Charge:0 Multiplicity: 1

C	0.82899000	1.22010500	-0.43143200
C	1.52321600	-0.07072700	-0.75403500
C	3.01809500	0.27644100	-0.93594900
C	3.12877500	1.76357100	-0.57402600
N	1.76337900	2.24154800	-0.32746600
O	4.05336800	2.51570000	-0.51202900
O	-0.39984800	1.31573100	-0.27858800
N	3.75189800	-1.95761800	0.00011500
C	4.77810600	-2.46393600	0.79357400
C	5.60838700	-1.40171400	1.16904200
H	4.85619100	-3.50900500	1.03351100
C	5.07847400	-0.21500000	0.59844700
H	6.48845200	-1.46724500	1.77573700
C	-4.32783000	-1.97536600	-0.27327100
C	-2.03895400	-1.91986600	0.69055200
H	-4.50250100	-0.92527100	0.03063200
H	-4.95140100	-2.16691700	-1.16249400
C	-2.15606300	-2.93579700	1.83124400

H	-0.94787000	-1.86667100	0.38360000
H	-2.29824400	-0.89636400	1.05184300
H	-1.60687600	-2.55269100	2.71708200
H	-3.20029100	-3.15729900	2.12177200
N	-2.86336700	-2.23478900	-0.55280000
C	-2.69551300	-3.66449100	-1.00669800
H	-2.93762000	-4.38675000	-0.19913300
H	-3.30264300	-3.88955000	-1.89240100
H	-1.62482600	-3.88019200	-1.25906300
C	-2.39237400	-1.31946200	-1.66513800
H	-3.00185500	-1.43032800	-2.57379500
H	-2.41549300	-0.25542200	-1.35716700
H	-1.33682600	-1.56088200	-1.93408100
H	-4.69822100	-2.62346200	0.54036500
O	-1.63418500	-4.21089800	1.47764800
H	2.98466200	-2.49672300	-0.39845000
Cl	0.61487800	-3.03616800	-0.75138000
H	-0.70317700	-4.13048500	1.11109800
Zn	-1.68596300	2.73833300	0.24665700
Cl	-0.66331500	4.61291800	0.33665700
Cl	-3.44760900	1.52037400	0.43205400
H	5.49122700	0.77082800	0.69111800
C	3.92699500	-0.57116100	-0.11638700
H	1.36228100	-0.81051600	0.07616900
H	3.30955900	0.16706700	-2.01862100
H	1.09449200	-0.59529000	-1.63722800
H	1.55277600	3.21822300	-0.08557300

Transition state d)TS1

	(Hartree/Particle)
Zero-point correction=	0.435827
Thermal correction to Energy=	0.486188
Thermal correction to Enthalpy=	0.487133
Thermal correction to Gibbs Free Energy=	0.346987
Sum of electronic and zero-point Energies=	-0.669342
Sum of electronic and thermal Energies=	-0.618981

Sum of electronic and thermal Enthalpies= -0.618037
Sum of electronic and thermal Free Energies= -0.758182

	E (Thermal)	CV	S
	KCal/Mol	Cal/Mol-Kelvin	Cal/Mol-Kelvin
Total	305.088	172.410	294.961

Charge:0 Multiplicity: 1

C	2.89007800	-1.23485300	0.26211300
C	3.49027500	-0.46221500	-0.79237200
C	4.69823600	0.07224500	-0.36516900
C	4.99586400	-0.53049900	1.02371400
N	3.83482500	-1.31771300	1.33649400
O	5.95469000	-0.42528200	1.73760300
O	1.80470900	-1.84757200	0.36826100
N	3.93260200	2.61199100	-0.90185400
C	4.96334400	3.38016800	-1.34593900
C	6.04290500	3.28974600	-0.41305900
H	4.93129700	3.95961000	-2.25766800
C	5.64232900	2.43928900	0.61105500
H	6.97916100	3.80605700	-0.50231200
C	-3.66534500	1.48218900	0.70585900
C	-3.24214900	2.67902900	1.56057500
H	-4.04334600	0.65598700	1.35777200
H	-2.75233000	1.07623200	0.19038000
H	-2.27331500	2.46142900	2.05587100
H	-3.13697800	3.62496600	0.99187100
N	-4.70782600	1.80565100	-0.36372200
C	-5.85708700	2.59140000	0.22696200
H	-5.51311300	3.55950300	0.64712500
H	-6.64034900	2.79445000	-0.51788500
H	-6.30728700	2.05038900	1.08054800
C	-5.24988500	0.51093000	-0.93044900
H	-5.98896200	0.68629100	-1.72552700
H	-4.43818500	-0.11906100	-1.35948100
H	-5.72020000	-0.11481400	-0.14646600
O	-4.25702400	2.97604700	2.52856800
H	3.00594300	2.46371700	-1.38114000
Cl	-0.98960100	2.96603200	-0.52274600
H	-4.18368200	2.38312000	3.30444000

H	6.21034800	2.14461800	1.47875100
C	4.32719700	1.93688700	0.29354800
H	3.56200900	1.66982900	1.03049600
H	5.52233600	0.37064800	-1.00820600
H	3.01398300	-0.31660200	-1.74884200
Zn	-0.03441500	-1.18980200	-0.49551600
Zn	-1.77067100	-1.74083500	0.09850500
Cl	-0.36059200	-3.68530000	0.05757900
Cl	-0.89730400	-0.23965000	1.83788200
Cl	-1.90401800	-0.54557600	-2.07581700
Cl	-3.93775100	-2.04495700	0.62077300
O	0.81030200	-1.69454700	-2.38316600
H	0.29391300	-1.57432900	-3.18521300
O	0.58346300	0.74345200	-0.50230300
H	1.19414500	1.00049300	0.24373200
O	1.47785300	-1.43418900	3.03768200
H	0.72059100	-1.41204900	3.62772800
O	1.91789400	-3.95771300	-1.82722500
H	2.09595500	-3.65393600	-0.91482300
O	1.74603100	1.11866800	1.91113600
H	1.67681900	0.22331700	2.34268300
O	1.64182600	2.19003900	-2.32482500
H	1.25310200	1.34221900	-1.95080500
H	0.96047400	2.85824200	-2.18616900
H	1.29406200	-2.58991300	-2.45756300
H	1.20362500	-1.98436200	2.27598800
H	1.22011100	-4.62096400	-1.75069000
H	1.02761700	1.63846800	2.27518600
H	-0.24311900	1.63425000	-0.46053300
C	-4.06178800	2.59828100	-1.47719900
H	-3.20535100	2.03691500	-1.91944900
H	-4.77018600	2.83637200	-2.28240800
H	-3.61954300	3.54321700	-1.10222700
H	3.64157100	-1.74084100	2.24466500

Intermediate d)Int1

	(Hartree/Particle)
Zero-point correction=	0.438226
Thermal correction to Energy=	0.487714
Thermal correction to Enthalpy=	0.488659
Thermal correction to Gibbs Free Energy=	0.352835
Sum of electronic and zero-point Energies=	-0.692394
Sum of electronic and thermal Energies=	-0.642906
Sum of electronic and thermal Enthalpies=	-0.641962
Sum of electronic and thermal Free Energies=	-0.777785

	E (Thermal)	CV	S
	KCal/Mol	Cal/Mol-Kelvin	Cal/Mol-Kelvin
Total	306.045	171.607	285.864

Charge:0 Multiplicity: 1

C	3.02277600	-1.11158100	0.60110400
C	3.79151600	-0.40956700	-0.28588300
C	4.74720600	0.47935300	0.45303200
C	4.55937300	0.04576600	1.93780400
N	3.51303600	-0.89646500	1.95191500
O	5.16506000	0.44619500	2.90328400
O	2.00012700	-1.89374800	0.45992200
N	3.01400100	2.13383000	-0.33523100
C	3.10565000	2.93022900	-1.38291100
C	4.50481900	3.37620600	-1.55181200
H	2.25728500	3.19345400	-2.04643500
C	5.25397400	2.80133000	-0.58514800
H	4.81209900	4.03631800	-2.34735000
C	-4.52605100	0.78456500	1.01161600
C	-3.98161700	1.34965600	2.32704300
H	-5.58994900	0.48225100	1.13186000
H	-3.97288300	-0.16956700	0.77675800
H	-3.84279900	0.53346100	3.06351000

H	-3.01232900	1.87968500	2.21351600
N	-4.40881600	1.73627800	-0.17782200
C	-5.37951500	2.88429500	-0.00525100
H	-5.25527700	3.35060700	1.00108000
H	-5.21595700	3.67072300	-0.76052300
H	-6.42606000	2.54898600	-0.07835800
C	-4.75355100	0.97462600	-1.44054800
H	-4.81308600	1.64113600	-2.31574500
H	-3.97475900	0.20789800	-1.67571000
H	-5.70517300	0.42541800	-1.34981400
O	-4.86212000	2.35655700	2.83838500
H	2.12907800	1.52249000	-0.02784800
Cl	-0.18194700	3.21522000	-0.68088800
H	-5.58630000	1.95769300	3.36295100
H	6.31333100	2.88696400	-0.40247000
C	4.36087900	1.96914600	0.32294800
H	4.31783800	2.45097900	1.34529800
H	5.80371000	0.31499400	0.14600300
H	3.73716300	-0.43907300	-1.35148000
Zn	0.29124000	-1.07016000	-0.39423200
Zn	-1.52983700	-1.71140000	-0.40559900
Cl	-0.10247200	-3.61742600	-0.25093200
Cl	-1.43185500	-0.46943200	1.69149000
Cl	-1.21519100	-0.10991500	-2.25019700
Cl	-3.71011900	-2.25602700	-0.64506400
O	1.38944300	-1.25030500	-2.27165800
H	0.89909700	-1.55959100	-3.04945400
O	0.77424200	0.83697400	0.22246700
H	0.72138700	0.97639400	1.23151200
O	0.90319000	-2.02440800	2.97123500
H	-0.02049500	-2.27751300	3.03006400
O	2.59297300	-3.41879200	-1.68669200
H	2.59143000	-3.24532500	-0.70609500
O	0.95483800	0.73006000	2.84928200

H	1.01753400	-0.26996100	2.89824200
O	1.24968900	1.74884000	-3.00117800
H	1.32831200	0.81472600	-2.76591500
H	0.32490500	1.98410100	-2.89107300
H	2.16372200	-1.94505300	-2.15749100
H	1.23127300	-2.39959900	2.11758800
H	1.88305400	-4.05226700	-1.83335700
H	0.15211900	0.95298700	3.31775200
H	0.17375100	1.83818000	-0.23745900
C	-3.00642700	2.28354800	-0.30816500
H	-2.24741200	1.47514300	-0.23844700
H	-2.84030900	2.78192300	-1.28122300
H	-2.76259200	3.02134300	0.47854600
H	3.11187800	-1.31613700	2.78618000

Transition state d)TS2

	(Hartree/Particle)
Zero-point correction=	0.440218
Thermal correction to Energy=	0.490536
Thermal correction to Enthalpy=	0.491480
Thermal correction to Gibbs Free Energy=	0.352664
Sum of electronic and zero-point Energies=	-0.722043
Sum of electronic and thermal Energies=	-0.671725
Sum of electronic and thermal Enthalpies=	-0.670781
Sum of electronic and thermal Free Energies=	-0.809597

	E (Thermal)	CV	S
	KCal/Mol	Cal/Mol-Kelvin	Cal/Mol-Kelvin
Total	307.816	173.902	292.163

Charge:0 Multiplicity: 1

C	3.18766500	-1.11089800	0.17045700

C	3.55134900	-0.23683200	-0.81680500
C	4.62776800	0.69574500	-0.32007300
C	4.97553300	0.12320000	1.07712300
N	4.02926200	-0.91478100	1.33203600
O	5.83017100	0.43691400	1.86671300
O	2.25020900	-1.99722300	0.26406600
N	3.28014500	2.67822600	-1.14225600
C	2.94958800	3.97047300	-0.71812000
C	3.59653100	4.20403100	0.49472000
H	2.28885700	4.60418400	-1.28186400
C	4.33474800	3.03272800	0.82969700
H	3.55437700	5.10163000	1.07722900
C	-4.38079100	1.09443600	1.06357900
C	-3.71612600	1.95198800	2.14557100
H	-5.43392100	0.87006100	1.34546400
H	-3.86700400	0.09304900	1.02626700
H	-3.52020900	1.34344000	3.05028600
H	-2.75374500	2.40709900	1.82683900
N	-4.36255200	1.71892300	-0.33097100
C	-5.32884800	2.88318700	-0.37148200
H	-5.13286300	3.58016600	0.47847100
H	-5.23351500	3.45979000	-1.30599300
H	-6.37435800	2.54846200	-0.27907900
C	-4.79358800	0.67285100	-1.33786000
H	-5.01620200	1.11684400	-2.32061100
H	-3.98978300	-0.08579000	-1.50301800
H	-5.67623100	0.10378100	-0.99882000
O	-4.54430800	3.07975400	2.45164800
H	2.94467700	2.22986500	-1.98337000
Cl	-0.22757100	3.01276400	-1.65138700
H	-5.19542600	2.86013500	3.14887000
H	4.96133200	2.90470300	1.69176800
C	4.12707800	2.09610800	-0.18648100
H	2.27516900	1.62535500	1.94316900

H	5.53956200	0.66426600	-0.96659600
H	3.20954200	-0.22463800	-1.82943500
Zn	0.27106600	-1.37697800	-0.05123100
Zn	-1.60564400	-1.81266900	0.02952300
Cl	-0.24899100	-3.74951900	0.59625300
Cl	-1.30479300	-0.03059900	1.68877200
Cl	-1.25100300	-0.60165600	-2.07791400
Cl	-3.81235100	-2.27817600	0.02900000
O	1.18862200	-1.95415800	-2.02906700
H	0.64431700	-2.41718600	-2.68065000
O	0.72510200	0.72796600	-0.28727100
H	1.69891600	0.86779900	-0.48922200
O	0.96089500	-1.57188500	2.25412800
H	0.29578300	-1.93071900	2.86225700
O	2.37997100	-4.06507900	-1.24432900
H	2.63550200	-3.67553200	-0.36954900
O	1.34357400	1.38507300	2.03628000
H	1.31880200	0.47069700	2.38440300
O	1.27733800	0.88102600	-2.70381500
H	1.39465200	-0.08129600	-2.49315300
H	0.67616500	0.90779300	-3.45494100
H	1.98521800	-2.59959200	-1.84118400
H	1.66578900	-2.25497100	2.17741900
H	1.57264100	-4.56240600	-1.07238000
H	0.60756200	1.28543200	0.59541400
H	0.26993300	1.74616200	-1.59842700
C	-2.98122700	2.20590100	-0.70324600
H	-2.20955900	1.42530800	-0.51785900
H	-2.91380300	2.46833300	-1.77435700
H	-2.67681800	3.09614400	-0.12244600
H	4.03340300	-1.51002000	2.14505400

Transition state d)TS3

	(Hartree/Particle)
Zero-point correction=	0.439039
Thermal correction to Energy=	0.487393
Thermal correction to Enthalpy=	0.488337
Thermal correction to Gibbs Free Energy=	0.356491
Sum of electronic and zero-point Energies=	-0.683625
Sum of electronic and thermal Energies=	-0.635271
Sum of electronic and thermal Enthalpies=	-0.634327
Sum of electronic and thermal Free Energies=	-0.766174

	E (Thermal)	CV	S
	KCal/Mol	Cal/Mol-Kelvin	Cal/Mol-Kelvin
Total	305.844	169.532	277.494

Charge:0 Multiplicity: 1

C	-3.17732600	-1.21139300	-0.18760600
C	-3.58580700	-0.30834200	0.75924400
C	-4.46045300	0.74247900	0.13901300
C	-4.74213800	0.17487900	-1.28035500
N	-3.90350500	-0.95459800	-1.42851700
O	-5.47997100	0.60115200	-2.13676400
O	-2.30608900	-2.15491500	-0.20304200
N	-3.02633200	2.58276000	1.16125400
C	-3.03881600	3.92362000	1.12121700
C	-3.83692000	4.35450500	-0.00764800
H	-2.50954600	4.56198300	1.82645300
C	-4.27434300	3.23465400	-0.67268400
H	-4.03182300	5.38480700	-0.24698300
C	4.32331500	1.26579900	-0.98965400
C	3.65296900	2.11734300	-2.07229700
H	5.38782300	1.07601500	-1.25008700
H	3.83464000	0.25033900	-0.97984900
H	3.46981700	1.50914100	-2.97976500
H	2.68303100	2.55705700	-1.75278400
N	4.25095600	1.86707200	0.41341100
C	5.14470800	3.08596400	0.48680500

H	4.91899900	3.78413200	-0.35396900
H	4.99935700	3.63938800	1.42888100
H	6.20946100	2.81687700	0.40502500
C	4.72961300	0.83222900	1.40974300
H	4.82261700	1.25421900	2.42281500
H	4.00940100	-0.01982600	1.48447300
H	5.69642100	0.38921700	1.11953500
O	4.46198200	3.26013700	-2.37404200
H	-2.52402400	1.97813400	1.83638700
Cl	-0.03715600	2.88711500	1.24492300
H	5.14833000	3.04016700	-3.03603900
H	-4.90051000	3.17107700	-1.55276500
C	-3.69277500	2.04511300	-0.02695100
H	-2.66528400	1.73347700	-0.85236000
H	-5.41311400	0.89685700	0.69146400
H	-3.36548300	-0.33232900	1.80441000
Zn	-0.24888900	-1.49141800	0.07463700
Zn	1.65914300	-1.79060200	-0.07712600
Cl	0.41868300	-3.78345700	-0.70378100
Cl	1.23174400	0.06715400	-1.63765100
Cl	1.32730500	-0.74726700	2.10848100
Cl	3.88960800	-2.15198400	-0.17651900
O	-1.11072800	-2.12578300	2.03822500
H	-0.54633200	-2.63047600	2.64204100
O	-0.80733000	0.49330600	0.30871300
H	-1.57518000	0.54413700	0.95469300
O	-0.98640700	-1.61998100	-2.16268400
H	-0.30979600	-1.80093600	-2.83541000
O	-2.41999600	-4.18301500	1.26199000
H	-2.92475600	-3.74145000	0.53666500
O	-1.61607500	1.51416700	-1.72233300
H	-1.55827000	0.51688200	-1.80459700
O	-1.39830800	0.70813000	2.79451400
H	-1.31414800	-0.26921100	2.81040000
H	-0.51680000	1.05745000	2.98532900
H	-1.92923000	-2.74232100	1.86277900
H	-1.58927600	-2.39495200	-2.15606200
H	-1.62824700	-4.52972000	0.82537800
H	-0.82850200	1.71438700	-1.12480800
H	-0.15831500	1.35447200	0.72682000

C	2.83561600	2.26122700	0.76405200
H	2.13316900	1.41203900	0.61517800
H	2.72971000	2.57180200	1.81804700
H	2.46393000	3.09729400	0.14045700
H	-3.84910100	-1.52674000	-2.25669900

Succinimide + ChCl + 2 $ZnCl_2$ • $3H_2O$ d)P

	(Hartree/Particle)
Zero-point correction=	0.440893
Thermal correction to Energy=	0.490659
Thermal correction to Enthalpy=	0.491603
Thermal correction to Gibbs Free Energy=	0.353616
Sum of electronic and zero-point Energies=	-0.743254
Sum of electronic and thermal Energies=	-0.693488
Sum of electronic and thermal Enthalpies=	-0.692544
Sum of electronic and thermal Free Energies=	-0.830531

	E (Thermal)	CV	S
	KCal/Mol	Cal/Mol-Kelvin	Cal/Mol-Kelvin
Total	307.893	171.366	290.418

Charge:0 Multiplicity: 1

C	4.00342500	-1.53481800	0.69753400
C	4.36485000	-0.76757800	-0.54760700
C	5.20274000	0.44448100	-0.08611900
C	5.36764800	0.26861800	1.43531100
N	4.60484800	-0.90576300	1.80499700
O	5.99793000	0.88861800	2.24171000
O	3.30977900	-2.53672700	0.79708600
N	3.26011500	2.09985300	-0.12991800
C	3.05656100	3.42484200	-0.51569000
C	4.25739500	3.92248700	-1.02993100

H	2.11009500	3.92749000	-0.39103300
C	5.22399300	2.88487600	-0.95512900
H	4.42414500	4.91049500	-1.40863800
C	-4.80662500	0.71799400	0.88296000
C	-4.33652800	1.36253900	2.19119300
H	-5.87107600	0.40506500	0.96895100
H	-4.22992800	-0.23716400	0.72632100
H	-4.22923200	0.59020500	2.97831500
H	-3.36720300	1.89681700	2.09854200
N	-4.64674500	1.60805600	-0.34897500
C	-5.64573800	2.74309800	-0.28879600
H	-5.57717400	3.26982200	0.69283700
H	-5.46019700	3.48761400	-1.08112600
H	-6.68169900	2.38359300	-0.39067200
C	-4.91797500	0.76944300	-1.58065700
H	-4.88666200	1.37554100	-2.50117500
H	-4.14982000	-0.03223700	-1.70323500
H	-5.89374100	0.25936600	-1.53036700
O	-5.25173800	2.38483700	2.59840600
H	2.54523700	1.47941000	0.25708100
Cl	-0.39395000	3.05003000	-0.72868600
H	-5.99818600	2.00739600	3.10776500
H	6.24656100	2.95593000	-1.26409700
C	4.59676400	1.76347100	-0.39624900
H	6.22509000	0.40378000	-0.54972200
H	3.43088900	-0.46358800	-1.09429000
Zn	0.00093200	-0.87359100	-0.07266700
Zn	-1.69070800	-1.76115700	-0.31615000
Cl	0.03683100	-3.44150500	-0.33520600
Cl	-1.73738100	-0.50017500	1.83927000
Cl	-1.38397700	-0.22028300	-2.15833500
Cl	-3.81323600	-2.47866700	-0.49286000
O	1.49366200	-0.79421500	-1.53128000
H	1.37120700	-0.00168300	-2.19308200

O	0.69375100	0.88701700	0.55063600
H	0.63328300	1.10034100	1.52040800
O	0.84998100	-1.82423800	1.85144100
H	0.26381400	-2.50798100	2.23553200
O	2.47760200	-3.07373200	-1.94927900
H	2.64647800	-3.11981900	-0.98306000
O	0.67004700	0.67245100	3.21310900
H	0.77081500	-0.27912600	3.01791900
O	1.15922500	1.45828100	-2.82862100
H	1.61248400	2.11254700	-2.27273500
H	0.21832400	1.69737700	-2.78730500
H	1.64952500	-1.61488300	-2.10279500
H	1.70725900	-2.30556100	1.67490400
H	1.89112200	-3.80712500	-2.15408800
H	-0.21702000	0.78188300	3.55666300
H	0.19765600	1.87802400	0.00967000
C	-3.25011000	2.17806400	-0.43963800
H	-2.47928600	1.41259200	-0.21338300
H	-3.01469200	2.55660500	-1.45228000
H	-3.08180200	3.01465300	0.26343400
H	4.90245800	-1.40293300	-1.27848800
H	4.51584400	-1.23024700	2.76514300

Structures for the proposed reactions between Pirrol et N-Phenyl Maleimide

Transition state a) TS1

	(Hartree/Particle)
Zero-point correction=	0.217556
Thermal correction to Energy=	0.232344
Thermal correction to Enthalpy=	0.233288
Thermal correction to Gibbs Free Energy=	0.173763
Sum of electronic and zero-point Energies=	0.257961
Sum of electronic and thermal Energies=	0.272749
Sum of electronic and thermal Enthalpies=	0.273693
Sum of electronic and thermal Free Energies=	0.214167

	E (Thermal)	CV	S
	KCal/Mol	Cal/Mol-Kelvin	Cal/Mol-Kelvin
Total	145.798	57.304	125.283

Charge:0 Multiplicity: 1

C	0.29921700	-1.13295900	-1.01817900
C	1.57427200	-0.59156300	-1.37426500
C	1.68524500	0.77429400	-1.04383100
C	0.27588100	1.21538400	-0.58339100
N	-0.51227900	0.03800000	-0.51137900
O	-0.04649700	2.34193000	-0.28083100
O	-0.16743100	-2.24462300	-1.02507300
C	-1.89831500	0.00189500	-0.12862700
C	-2.51715800	-1.23519300	0.13623900
C	-2.63360300	1.19585100	0.00015700
C	-3.85612300	-1.26584100	0.52597100
H	-1.96505500	-2.17232500	0.01765200
C	-3.97125800	1.14328000	0.39467100
H	-2.16929400	2.16390100	-0.20536300
C	-4.58865300	-0.08208700	0.66107800

H	-4.33303300	-2.22533100	0.72394700
H	-4.53628900	2.06974400	0.49217300
H	-5.63106200	-0.11543100	0.96610100
N	2.13525600	0.02716200	1.41545500
C	2.96110100	-1.05086700	1.39873000
C	4.14016700	-0.72202000	0.64620800
H	2.75877800	-1.98326300	1.91502200
C	3.97214700	0.54470100	0.12403100
H	4.98754500	-1.37537300	0.52929200
H	1.20357700	0.06395900	1.81155000
H	4.66373000	1.10307000	-0.48403700
C	2.62496400	1.01644000	0.46274100
H	2.43273700	2.07135800	0.70728700
H	2.21595000	1.47988700	-1.69370100
H	2.31570400	-1.18268100	-1.87042600

Intermediate a) Int1

	(Hartree/Particle)
Zero-point correction=	0.218996
Thermal correction to Energy=	0.233905
Thermal correction to Enthalpy=	0.234849
Thermal correction to Gibbs Free Energy=	0.173900
Sum of electronic and zero-point Energies=	0.258466
Sum of electronic and thermal Energies=	0.273375
Sum of electronic and thermal Enthalpies=	0.274320
Sum of electronic and thermal Free Energies=	0.213371

	E (Thermal)	CV	S
	KCal/Mol	Cal/Mol-Kelvin	Cal/Mol-Kelvin
Total	146.777	57.825	128.278

Charge:0 Multiplicity: 1

C	-0.04458000	1.50070200	0.60820500
C	1.28732100	1.19954400	0.91308600
C	1.56621500	-0.25034500	0.85165700
C	0.18430500	-0.88163100	0.52050300
N	-0.73659000	0.16172000	0.35072100
O	0.01464700	-2.07772400	0.37721900
O	-0.67491900	2.52668900	0.47315500
C	-2.12868900	-0.02805700	0.04723600
C	-2.92191300	1.07681400	-0.32161400
C	-2.70603800	-1.31177900	0.10741300
C	-4.26997900	0.88930800	-0.62292500
H	-2.48930000	2.08289300	-0.35618200
C	-4.05697400	-1.47868600	-0.20047700
H	-2.10523500	-2.18027700	0.38960100
C	-4.84537800	-0.38483200	-0.56725000
H	-4.87885100	1.74782400	-0.90298600
H	-4.49652100	-2.47375200	-0.15161100
H	-5.89623000	-0.52127200	-0.80432000
N	3.07706600	0.74424400	-0.84533200
C	4.40085400	0.72261400	-0.83527900
C	4.86635800	-0.58967900	-0.36767000
H	5.05358200	1.54943000	-1.12048500
C	3.78129000	-1.34075900	-0.04711900
H	5.91129900	-0.84910400	-0.29082000
H	2.41662500	1.54986700	-0.92729300
H	3.73973200	-2.35054900	0.34118200
C	2.52276600	-0.56304700	-0.34570900
H	1.94335000	-1.06711000	-1.17457100
H	1.97404200	-0.67526800	1.79131500
H	1.91300800	1.89285200	1.42510500

Transition state a)TS2

	(Hartree/Particle)
Zero-point correction=	0.216333
Thermal correction to Energy=	0.230890
Thermal correction to Enthalpy=	0.231834
Thermal correction to Gibbs Free Energy=	0.173064
Sum of electronic and zero-point Energies=	0.279040
Sum of electronic and thermal Energies=	0.293597
Sum of electronic and thermal Enthalpies=	0.294541
Sum of electronic and thermal Free Energies=	0.235771

	E (Thermal)	CV	S
	KCal/Mol	Cal/Mol-Kelvin	Cal/Mol-Kelvin
Total	144.886	57.046	123.693

Charge:0 Multiplicity: 1

C	0.19405800	1.85025600	-0.15884900
C	-1.15933600	1.83505900	-0.55024900
C	-1.59184100	0.46650700	-0.99257100
C	-0.31702200	-0.39933500	-0.83959600
N	0.70224200	0.42621400	-0.33180400
O	-0.26418000	-1.58758300	-1.08402000
O	0.93436100	2.69173900	0.29474500
C	2.04616700	-0.01956300	-0.07622600
C	2.89667000	0.75652200	0.73556500
C	2.51426000	-1.22915900	-0.62517200
C	4.19705300	0.32019900	0.98639600
H	2.54955400	1.70757300	1.15267800
C	3.81807400	-1.65001100	-0.35894500
H	1.86440900	-1.84467400	-1.25181700
C	4.66467700	-0.88225200	0.44536700
H	4.85284900	0.92629200	1.61009900
H	4.17477900	-2.58623600	-0.78572700

H	5.67886000	-1.21462500	0.64703900
N	-4.03066400	0.28492700	-0.21891500
C	-4.75789800	-0.61225100	0.47951500
C	-3.86722300	-1.45441300	1.23202200
H	-5.84263400	-0.67529200	0.46307600
C	-2.57343300	-1.05397400	0.97159500
H	-4.18790000	-2.26877100	1.86153100
H	-4.36814300	0.99959900	-0.85042400
H	-1.65134100	-1.48619800	1.34390900
C	-2.61118500	0.12748000	0.10208300
H	-2.19454300	1.18841300	0.73488400
H	-1.99222300	0.38566800	-2.01938100
H	-1.66428500	2.72663000	-0.84223900

N-Phenyl succinimide a)P

	(Hartree/Particle)
Zero-point correction=	0.220618
Thermal correction to Energy=	0.235546
Thermal correction to Enthalpy=	0.236490
Thermal correction to Gibbs Free Energy=	0.175941
Sum of electronic and zero-point Energies=	0.170640
Sum of electronic and thermal Energies=	0.185568
Sum of electronic and thermal Enthalpies=	0.186512
Sum of electronic and thermal Free Energies=	0.125963

	E (Thermal)	CV	S
	KCal/Mol	Cal/Mol-Kelvin	Cal/Mol-Kelvin
Total	147.807	57.723	127.436

Charge:0 Multiplicity: 1

C	-0.26273900	1.78418100	-0.14107900
C	1.17718400	1.96449500	0.29470800

C	1.61652600	0.64023400	0.94747600
C	0.35309300	-0.24001300	0.97177200
N	-0.69309200	0.47336400	0.27811800
O	0.22181000	-1.32369400	1.47032200
O	-0.94930300	2.57441900	-0.73377700
C	-2.02685200	-0.05001500	0.06653300
C	-3.10389000	0.83306700	-0.12455100
C	-2.23320300	-1.44078400	0.04879200
C	-4.38282600	0.31482600	-0.33713400
H	-2.95092700	1.91474400	-0.12317900
C	-3.52113300	-1.93770500	-0.16040500
H	-1.40634400	-2.13415000	0.21613600
C	-4.59739700	-1.06673300	-0.35560200
H	-5.21857000	0.99799800	-0.48898800
H	-3.68400400	-3.01548000	-0.16954600
H	-5.59749300	-1.46219100	-0.51969800
N	2.60804700	-0.53547500	-1.07830500
C	3.83964900	-1.07297900	-1.46491600
C	4.73853000	-0.89101100	-0.41329500
H	3.99249000	-1.53302300	-2.42367400
C	4.04809100	-0.23550500	0.64518700
H	5.76747600	-1.19101500	-0.39647300
H	1.76815700	-0.54291300	-1.62423700
H	4.46870600	0.03670600	1.59204300
C	2.73242500	-0.02214100	0.22427700
H	1.81605700	2.24420400	-0.56906100
H	1.93252800	0.81382400	2.01144500
H	1.26243800	2.82077900	0.99360300

Transition state b) TS1

	(Hartree/Particle)
Zero-point correction=	0.220538
Thermal correction to Energy=	0.241426

Thermal correction to Enthalpy=	0.242370
Thermal correction to Gibbs Free Energy=	0.165408
Sum of electronic and zero-point Energies=	0.143585
Sum of electronic and thermal Energies=	0.164474
Sum of electronic and thermal Enthalpies=	0.165418
Sum of electronic and thermal Free Energies=	0.088456

	E (Thermal)	CV	S
	KCal/Mol	Cal/Mol-Kelvin	Cal/Mol-Kelvin
Total	151.497	73.834	161.981

Charge:0 Multiplicity: 1

C	0.09797900	0.11250100	0.65295900
C	-1.06323600	-0.56299300	1.15478000
C	-2.18127000	0.26091500	1.07225300
C	-1.66434700	1.67117700	0.71906400
N	-0.25659600	1.50759500	0.43761200
O	-2.28684000	2.69666000	0.66714900
O	1.24787500	-0.28185500	0.39928600
C	0.64274600	2.56672600	0.03925200
C	0.27929800	3.90584700	0.26814700
C	1.86813000	2.25518600	-0.57448800
C	1.14826000	4.92569900	-0.12486800
H	-0.67154100	4.15971800	0.74145300
C	2.72406800	3.29066100	-0.95494500
H	2.17100000	1.21759000	-0.74408200
C	2.36954100	4.62529300	-0.73544000
H	0.86778100	5.96443100	0.05036900
H	3.67799500	3.04949400	-1.42561900
H	3.04240800	5.42580500	-1.03595400
N	-3.15318500	-1.55769700	-0.62207300
C	-4.44280300	-1.91171800	-0.39071200
C	-5.23877700	-0.72319900	-0.31115600
H	-4.78618600	-2.93544400	-0.29820000

C	-4.39774300	0.36479500	-0.48866200
H	-6.30278700	-0.70876100	-0.15397100
H	-2.34368300	-2.17290500	-0.68879700
Zn	1.87229000	-2.15677500	0.03052900
Cl	3.99620500	-2.21938000	0.27360800
Cl	0.05083900	-3.22473400	-0.52584100
H	-4.66342500	1.41117800	-0.49094100
C	-3.03810900	-0.12256500	-0.61870900
H	-2.30496700	0.32978700	-1.29028400
H	-3.06343700	0.17106700	1.70493400
H	-1.03381200	-1.57644000	1.51807200

Intermediate b) Int1

	(Hartree/Particle)
Zero-point correction=	0.222805
Thermal correction to Energy=	0.243383
Thermal correction to Enthalpy=	0.244327
Thermal correction to Gibbs Free Energy=	0.169187
Sum of electronic and zero-point Energies=	0.122986
Sum of electronic and thermal Energies=	0.143564
Sum of electronic and thermal Enthalpies=	0.144508
Sum of electronic and thermal Free Energies=	0.069368

	E (Thermal)	CV	S
	KCal/Mol	Cal/Mol-Kelvin	Cal/Mol-Kelvin
Total	152.725	74.098	158.146

Charge:0 Multiplicity: 1

C	0.22051900	0.18821000	-1.14153500
C	1.54133400	0.17289000	-1.50487600
C	2.22395700	1.42787400	-1.09114600
C	1.07539200	2.29099800	-0.49075500

N	-0.10007600	1.50907200	-0.53875900
O	1.21729000	3.39180900	-0.00947500
O	-0.70503200	-0.67494100	-1.22411400
C	-1.41058800	1.95405700	-0.13246400
C	-1.53273800	3.00327900	0.79621600
C	-2.55536200	1.33536900	-0.66581400
C	-2.80557200	3.41528200	1.19646200
H	-0.64903800	3.50762800	1.19048500
C	-3.81729400	1.75732700	-0.24535300
H	-2.46653300	0.52488800	-1.39803100
C	-3.94851800	2.79433600	0.68446700
H	-2.90363300	4.22881600	1.91451300
H	-4.70589300	1.27102300	-0.64902200
H	-4.93634200	3.11660200	1.00582500
N	2.75753200	0.15609800	1.00392400
C	3.50716100	-0.93940700	0.98534600
C	4.64556200	-0.73699000	0.07720400
H	3.31865200	-1.85354100	1.56584600
C	4.52661900	0.48195200	-0.50135200
H	5.41038200	-1.48581600	-0.07731200
H	1.83543000	0.26740400	1.44956400
Zn	-0.95139300	-2.14400400	-0.02035900
Cl	-2.56756500	-3.52225100	-0.39015600
Cl	0.67484200	-1.94966300	1.50798100
H	5.17478400	0.95474900	-1.22607900
C	3.27968800	1.17517800	0.01428300
H	3.51878300	2.13738900	0.54974400
H	2.69597900	1.97086300	-1.94260500
H	2.02427100	-0.61059100	-2.04674500

Transition state b)TS2

	(Hartree/Particle)
Zero-point correction=	0.218570
Thermal correction to Energy=	0.239301
Thermal correction to Enthalpy=	0.240245
Thermal correction to Gibbs Free Energy=	0.162391
Sum of electronic and zero-point Energies=	0.154800
Sum of electronic and thermal Energies=	0.175530
Sum of electronic and thermal Enthalpies=	0.176474
Sum of electronic and thermal Free Energies=	0.098621

	E (Thermal)	CV	S
	KCal/Mol	Cal/Mol-Kelvin	Cal/Mol-Kelvin
Total	150.163	73.879	163.856

Charge:0 Multiplicity: 1

C	1.01001900	0.15826200	-0.38521200
C	-0.33737800	0.54222100	-0.23477200
C	-1.23721300	-0.55319500	-0.78043500
C	-0.24846900	-1.61523500	-1.31899700
N	1.05274500	-1.14518800	-1.01777700
O	-0.50001000	-2.66117300	-1.86307900
O	2.03701400	0.75810200	-0.02827600
N	-3.30639100	-0.71820800	0.76610000
C	-3.82984700	-1.66822800	1.57912100
C	-2.83014400	-2.66659400	1.83952200
H	-4.84506800	-1.65410600	1.96452800
C	-1.68306800	-2.31111500	1.16370800
H	-2.99042900	-3.54288400	2.44710600
Zn	3.96161000	0.46898100	0.11304900
Cl	4.33847800	-1.60876800	-0.34939900
Cl	4.79463000	2.34684800	0.70594400
H	-0.74407500	-2.84876900	1.12204100

C	-1.89567100	-1.01295500	0.51875900
H	-1.08489800	-0.12388100	1.12162800
H	-1.95172900	-0.24084600	-1.56543200
H	-0.64258200	1.56387100	-0.16555300
H	1.91012400	-1.65330000	-1.25413700
C	-4.01225200	0.43567800	0.25685200
C	-3.44925300	1.71374400	0.39018700
C	-5.25799700	0.24555900	-0.36562200
C	-4.15138400	2.81481500	-0.10749000
H	-2.47565100	1.85407600	0.86668900
C	-5.94337200	1.35967900	-0.85398100
H	-5.68004100	-0.75257000	-0.48416900
C	-5.39390400	2.64083400	-0.72556700
H	-3.72203300	3.81516100	-0.01313400
H	-6.91048800	1.22766400	-1.34330800
H	-5.93518100	3.50631300	-1.11283900

N_ Phenyl succinimide $ZnCl_2$ b)P

	(Hartree/Particle)
Zero-point correction=	0.222901
Thermal correction to Energy=	0.244124
Thermal correction to Enthalpy=	0.245068
Thermal correction to Gibbs Free Energy=	0.166084
Sum of electronic and zero-point Energies=	0.063491
Sum of electronic and thermal Energies=	0.084713
Sum of electronic and thermal Enthalpies=	0.085658
Sum of electronic and thermal Free Energies=	0.006673

	E (Thermal)	CV	S
	KCal/Mol	Cal/Mol-Kelvin	Cal/Mol-Kelvin
Total	153.190	74.522	166.237

Charge:0 Multiplicity: 1

C	0.44101000	-0.87794100	-0.50496200
C	1.79764200	-1.44321000	-0.86363800
C	2.73592100	-0.22857700	-1.02946000
C	1.90025800	0.98719800	-0.58710200
N	0.55564300	0.52291100	-0.34049000
O	2.25116600	2.12936900	-0.47050000
O	-0.57186000	-1.54923000	-0.37966400
C	-0.53822700	1.39923500	0.04944500
C	-1.03801100	1.33073700	1.35895800
C	-1.06266800	2.29304700	-0.89626800
C	-2.09593900	2.17344900	1.71557700
H	-0.61989500	0.63613400	2.09067100
C	-2.11451900	3.12963900	-0.51464200
H	-0.66524000	2.33240200	-1.90929200
C	-2.63116100	3.06952200	0.78472500
H	-2.50619300	2.12150200	2.72550400
H	-2.53857400	3.82672800	-1.23826200
H	-3.45784200	3.72014500	1.07059700
N	4.71694700	0.76577900	0.20445600
C	5.88334300	0.32145100	0.83021000
C	5.91682100	-1.07172900	0.74349300
H	6.58935100	0.99623100	1.27945300
C	4.74649800	-1.49542800	0.05474100
H	6.68540800	-1.71472500	1.12483400
H	4.41222500	1.72438500	0.12002100
Zn	-2.56285200	-1.22156400	-0.01810300
Cl	-3.25547400	-0.68890400	-1.95769200
Cl	-2.65197600	-1.62139300	2.07107500
H	4.49393500	-2.51002800	-0.17573400
C	4.00902900	-0.35062900	-0.26765300
H	2.14386900	-2.13648600	-0.06121800
H	2.98704400	-0.08894600	-2.11887600
H	1.74557100	-2.06787300	-1.77909900

Transition state c) TS1

	(Hartree/Particle)
Zero-point correction=	0.393616
Thermal correction to Energy=	0.427589
Thermal correction to Enthalpy=	0.428533
Thermal correction to Gibbs Free Energy=	0.322727
Sum of electronic and zero-point Energies=	0.162408
Sum of electronic and thermal Energies=	0.196380
Sum of electronic and thermal Enthalpies=	0.197324
Sum of electronic and thermal Free Energies=	0.091519

	E (Thermal)	CV	S
	KCal/Mol	Cal/Mol-Kelvin	Cal/Mol-Kelvin
Total	268.316	119.887	222.687

Charge:0 Multiplicity: 1

C	-0.15988700	0.80015200	-0.76147100
C	1.04336700	1.39667000	-1.29385900
C	1.05755700	2.75176100	-1.04644000
C	-0.28959000	3.13885300	-0.42499700
N	-0.96811800	1.86026400	-0.22052200
O	-0.76575800	4.20048800	-0.15079100
O	-0.48510300	-0.40054800	-0.74667400
C	-2.28410700	1.67720500	0.34144700
C	-2.45552600	0.74736200	1.39085100
C	-3.36858500	2.40548300	-0.17260300
C	-3.74964200	0.53642700	1.89585400
H	-1.59014200	0.30421600	1.90286600
C	-4.64265400	2.17977800	0.35266700
H	-3.21821100	3.13547400	-0.96865300
C	-4.83696200	1.24123200	1.37376300
H	-3.89809200	-0.17560300	2.71116400
H	-5.49423300	2.73608800	-0.04353200
H	-5.83920500	1.06251400	1.76295100

N	3.45007000	2.61082500	0.30229500
C	4.18706200	3.72617000	0.05799100
C	3.42193400	4.88344000	0.40981300
H	5.19419500	3.69936700	-0.33066800
C	2.18779700	4.43868200	0.87188700
H	3.76360700	5.89612700	0.33494700
C	1.11909200	-4.26172200	-0.72398200
C	1.76280000	-2.23590500	0.59720300
H	0.05740900	-3.94355700	-0.65895800
H	1.24620900	-4.81567800	-1.66751000
C	1.68497300	-3.02614300	1.90372100
H	2.55663100	-1.43877100	0.68045400
H	0.80596600	-1.67098100	0.44262600
H	1.32221900	-2.36859400	2.71897700
H	1.02072900	-3.91269300	1.85385800
N	2.05176700	-3.07361500	-0.64381100
C	3.48309000	-3.55837300	-0.61460100
H	3.68874900	-4.11977700	0.32290500
H	3.72001000	-4.19906400	-1.47488400
H	4.19216400	-2.69753000	-0.62757500
C	1.84160400	-2.21303300	-1.87094600
H	1.98025200	-2.78237300	-2.80116700
H	0.82641600	-1.76301100	-1.88075500
H	2.56520800	-1.36391800	-1.89037000
H	1.29444300	-4.96905900	0.10803500
O	2.95510900	-3.58489800	2.25184200
H	3.74616100	1.54393500	0.07593200
Cl	4.11702800	-0.06152800	-0.40529800
H	3.59077500	-2.87606400	2.50218400
Zn	-2.25319200	-1.31608200	-0.30294300
Cl	-3.70876000	-1.00483800	-1.87183500
Cl	-1.50933000	-2.74477600	1.19567700
H	1.36785300	5.03994100	1.22130100
C	2.16170200	3.00504400	0.75313600

H	1.55779400	2.32654300	1.33290000
H	1.66542100	3.50343100	-1.54345500
H	1.83731700	0.81975900	-1.74789700

Intermediate c) Int1

	(Hartree/Particle)
Zero-point correction=	0.395614
Thermal correction to Energy=	0.428663
Thermal correction to Enthalpy=	0.429608
Thermal correction to Gibbs Free Energy=	0.326806
Sum of electronic and zero-point Energies=	0.120982
Sum of electronic and thermal Energies=	0.154031
Sum of electronic and thermal Enthalpies=	0.154975
Sum of electronic and thermal Free Energies=	0.052174

	E (Thermal)	CV	S
	KCal/Mol	Cal/Mol-Kelvin	Cal/Mol-Kelvin
Total	268.990	118.542	216.364

Charge:0 Multiplicity: 1

C	-0.06990100	0.84767600	-0.68339600
C	1.20908200	1.21743500	-0.99265500
C	1.44854900	2.65512000	-0.65372800
C	0.08485300	3.13330600	-0.08642000
N	-0.77520000	2.00312800	-0.09760600
O	-0.20400900	4.23548100	0.31536100
O	-0.64789400	-0.27657000	-0.82147700
C	-2.15530900	1.96913900	0.30965600
C	-2.54672600	1.05384000	1.31534700
C	-3.09228800	2.81809300	-0.30311900
C	-3.90607000	0.98829100	1.67389200
H	-1.79170900	0.53223000	1.92582400

C	-4.43304100	2.73187300	0.07400400
H	-2.77255700	3.53156300	-1.06237100
C	-4.84325100	1.81416600	1.05015800
H	-4.22184500	0.29379200	2.45587700
H	-5.16910600	3.38125700	-0.40229600
H	-5.89597100	1.74428700	1.32031600
N	3.85824800	2.28055900	-0.02560300
C	4.75406800	3.24022000	-0.01900900
C	4.16536700	4.53461600	0.40219000
H	5.80063000	3.10825300	-0.28345400
C	2.85844800	4.32043900	0.66119700
H	4.73085700	5.44701600	0.46748100
C	0.50570200	-4.29371200	-0.72113500
C	1.57397800	-2.51418800	0.69114700
H	-0.48935600	-3.81638100	-0.56851600
H	0.48372400	-4.79066900	-1.70391000
C	1.42256000	-3.39803000	1.92931100
H	2.49318400	-1.88261500	0.76846700
H	0.71530400	-1.78068300	0.65796000
H	1.27003300	-2.76885500	2.82815600
H	0.57246200	-4.10967900	1.86255500
N	1.61622700	-3.27178800	-0.63742200
C	2.94863400	-3.96733800	-0.79028200
H	3.13000500	-4.65343000	0.06689400
H	3.00057400	-4.54794400	-1.72368000
H	3.78199200	-3.23661100	-0.80148400
C	1.42722800	-2.25651500	-1.74467600
H	1.09049300	-2.70440800	-2.68760300
H	0.64998900	-1.51599100	-1.39911700
H	2.34783200	-1.67546300	-1.93784600
H	0.61026500	-5.07445600	0.05414200
O	2.56273700	-4.24732300	2.09903000
H	4.03654100	0.85690600	-0.31003200
Cl	4.34978000	-0.51337200	-0.48866400

H	3.31905900	-3.74525500	2.47082900
Zn	-2.39521700	-0.97268100	-0.27146100
Cl	-4.01788900	-0.91783300	-1.73369000
Cl	-1.72865300	-2.44196600	1.29607300
H	2.08561200	5.01042500	0.97221100
C	2.54027500	2.84735300	0.41536300
H	2.24282400	2.32962500	1.36685100
H	1.69139000	3.24730700	-1.56932100
H	1.95469400	0.61739800	-1.46329200

Transition state c)TS2

	(Hartree/Particle)
Zero-point correction=	0.392348
Thermal correction to Energy=	0.425766
Thermal correction to Enthalpy=	0.426710
Thermal correction to Gibbs Free Energy=	0.321950
Sum of electronic and zero-point Energies=	0.169739
Sum of electronic and thermal Energies=	0.203157
Sum of electronic and thermal Enthalpies=	0.204101
Sum of electronic and thermal Free Energies=	0.099341

	E (Thermal)	CV	S
	KCal/Mol	Cal/Mol-Kelvin	Cal/Mol-Kelvin
Total	267.172	119.054	220.486

Charge:0 Multiplicity: 1

C	-0.28614900	0.64232100	-0.65002800
C	1.00494100	1.11328700	-0.95459400
C	0.99842500	2.63854100	-1.01449000
C	-0.44839600	3.01419000	-0.64194100
N	-1.14742600	1.76310500	-0.39387500

O	-0.97255800	4.08836700	-0.54970900
O	-0.63484100	-0.55752400	-0.53564600
C	-2.51783600	1.63420400	0.04608500
C	-2.78289300	0.94642600	1.24810100
C	-3.55378200	2.17291500	-0.73127700
C	-4.11640600	0.77700300	1.64461600
H	-1.96896500	0.62913500	1.90831500
C	-4.87392600	1.99711900	-0.30945800
H	-3.33124500	2.71975300	-1.64724400
C	-5.15702000	1.29375600	0.86713900
H	-4.33904100	0.24353300	2.57006000
H	-5.68905500	2.40545700	-0.90835500
H	-6.19121700	1.14725000	1.17642400
N	3.40274100	2.89020400	-0.14757900
C	4.01125400	3.64488300	0.78299200
C	3.02547700	4.28439400	1.62558200
H	5.08622900	3.73973400	0.87098800
C	1.78470200	3.86012600	1.20194100
H	3.25415800	4.96742400	2.42071800
C	1.43921300	-4.02136600	-0.52270900
C	2.06232100	-1.83395900	0.51540000
H	0.37851700	-3.79006300	-0.28244500
H	1.44709600	-4.63108600	-1.44040600
C	2.29665500	-2.49062100	1.87754900
H	2.74040200	-0.94957200	0.38814700
H	1.01070000	-1.42472500	0.49022900
H	2.02467400	-1.78341900	2.68666800
H	1.71153600	-3.42201700	2.02451400
N	2.25144500	-2.75632700	-0.68536100
C	3.71147900	-3.11005300	-0.84734800
H	4.11402200	-3.56262600	0.08588500
H	3.87599800	-3.81050600	-1.67859000
H	4.32328400	-2.20148400	-1.04742300
C	1.77679100	-2.02852900	-1.92439600

H	1.93003800	-2.62293700	-2.83694900
H	0.69629300	-1.77153800	-1.85038500
H	2.32468700	-1.06378200	-2.05637500
H	1.82654000	-4.64738400	0.30223100
O	3.65166400	-2.91823800	2.03311600
H	3.89793900	1.96825800	-0.77060500
Cl	4.33279100	0.45444300	-1.09564700
H	4.25271700	-2.14602700	2.13559900
Zn	-2.23779000	-1.56546900	0.01833400
Cl	-3.87602400	-1.68048900	-1.37823600
Cl	-1.15470000	-2.61058400	1.62294300
H	0.82186500	4.14961500	1.58746100
C	1.97811600	2.91796700	0.11220100
H	1.75787900	1.50812500	0.34465400
H	1.28838800	3.08384500	-1.98697400
H	1.73585900	0.55647300	-1.51676800

N-Phenyl succinimide + ChCl + $ZnCl_2$ c)P

	(Hartree/Particle)
Zero-point correction=	0.397703
Thermal correction to Energy=	0.431908
Thermal correction to Enthalpy=	0.432852
Thermal correction to Gibbs Free Energy=	0.324831
Sum of electronic and zero-point Energies=	0.093829
Sum of electronic and thermal Energies=	0.128034
Sum of electronic and thermal Enthalpies=	0.128978
Sum of electronic and thermal Free Energies=	0.020958

	E (Thermal)	CV	S
	KCal/Mol	Cal/Mol-Kelvin	Cal/Mol-Kelvin
Total	271.026	120.108	227.348

Charge:0 Multiplicity: 1

C	-0.36043000	0.66798200	-0.55662900
C	0.92661300	1.34718000	-0.91917100
C	0.61864200	2.85818100	-0.98531900
C	-0.82528700	3.00175500	-0.48327700
N	-1.33342000	1.63415800	-0.27096300
O	-1.51442500	3.95969000	-0.31164600
O	-0.50063500	-0.56134300	-0.50106600
C	-2.67228900	1.32284700	0.19600100
C	-2.82845300	0.62880800	1.41027200
C	-3.77658400	1.71325500	-0.57551400
C	-4.12305200	0.30161800	1.83244200
H	-1.96981600	0.40221800	2.04955200
C	-5.05840700	1.38310200	-0.12744000
H	-3.64030400	2.26361100	-1.50605900
C	-5.23218200	0.67295400	1.06593100
H	-4.26174000	-0.24141700	2.76905200
H	-5.92743500	1.67639600	-0.71851000
H	-6.23604100	0.40909800	1.39924100
N	2.94881500	3.45497600	-0.18764300
C	3.55619700	4.42569100	0.60517500
C	2.56280700	5.28766400	1.08277600
H	4.61802100	4.44760000	0.77324000
C	1.31605200	4.83280300	0.57936900
H	2.71123700	6.14059200	1.71330000
C	1.88634300	-4.22469300	0.05954000
C	2.37524500	-1.84373600	0.59136300
H	0.82127800	-4.06869400	0.32875000
H	1.92673700	-5.02615100	-0.69489400
C	3.15847700	-2.05236200	1.88298500
H	2.78114000	-0.86155600	0.09545500
H	1.29703300	-1.66474500	0.80388800
H	2.84644200	-1.29778800	2.63422500
H	3.04984800	-3.06180000	2.32160500

N	2.51714000	-2.94563900	-0.44311500
C	3.96981400	-3.19610800	-0.77629700
H	4.56157400	-3.42871700	0.13548900
H	4.09294900	-4.00558300	-1.50647500
H	4.44168400	-2.27270100	-1.19231000
C	1.80639900	-2.50001600	-1.70548700
H	1.79370200	-3.28899000	-2.47102900
H	0.76233000	-2.19358100	-1.49824800
H	2.31846900	-1.61021900	-2.14112500
H	2.39722700	-4.59040400	0.96805700
O	4.56655300	-1.93632600	1.66845100
H	3.42257700	2.67620500	-0.64029200
Cl	3.87400500	0.27066300	-0.99453100
H	4.77874900	-1.08218700	1.19826200
Zn	-2.05322900	-1.79818900	-0.07364000
Cl	-3.46272400	-1.82537400	-1.68172500
Cl	-1.09469400	-2.71408100	1.63371600
H	0.35912200	5.28493500	0.75610400
C	1.56674200	3.69386800	-0.19770700
H	1.70483300	1.11701500	-0.14188900
H	0.63972500	3.20871900	-2.05596000
H	1.38598000	0.95787800	-1.85499600

Transition state d) TS1

	(Hartree/Particle)
Zero-point correction=	0.511856
Thermal correction to Energy=	0.567034
Thermal correction to Enthalpy=	0.567978
Thermal correction to Gibbs Free Energy=	0.418222
Sum of electronic and zero-point Energies=	-0.544935
Sum of electronic and thermal Energies=	-0.489757

Sum of electronic and thermal Enthalpies= -0.488813
Sum of electronic and thermal Free Energies= -0.638569

	E (Thermal)	CV	S
	KCal/Mol	Cal/Mol-Kelvin	Cal/Mol-Kelvin
Total	355.819	192.064	315.187

Charge:0 Multiplicity: 1

C	2.58592000	-0.04137700	-0.72246000
C	2.60008400	1.24020000	-1.36955800
C	3.75941000	1.93237600	-1.06120000
C	4.67014600	0.97182500	-0.27748200
N	3.89362600	-0.24405500	-0.12121900
O	5.78640200	1.13709300	0.12971300
O	1.72938500	-0.94061800	-0.63373300
C	4.34127400	-1.44404200	0.53747200
C	3.76335800	-2.68099300	0.19888500
C	5.35928800	-1.37330200	1.50506700
C	4.20018100	-3.84046400	0.84304800
H	2.97214200	-2.74512200	-0.55468300
C	5.78568800	-2.54508500	2.13191000
H	5.82002200	-0.41694700	1.76138800
C	5.20955300	-3.77823300	1.80784100
H	3.74752900	-4.79915000	0.58868800
H	6.57415600	-2.49397800	2.88341300
H	5.54524300	-4.68513400	2.30638000
N	2.45008200	4.11902900	-0.20912400
C	3.14769100	5.26745000	-0.42337600
C	4.44355700	5.13632500	0.16461700
H	2.74966300	6.12287900	-0.95036000
C	4.52109000	3.86946600	0.73316200
H	5.20011100	5.89712800	0.16463100
C	-4.38620800	0.59437000	1.09270800
C	-4.20547400	1.73305900	2.09892000

H	-4.42350600	-0.38862800	1.62461600
H	-3.47558800	0.55684800	0.43467000
H	-3.15774400	1.74592400	2.46527200
H	-4.44358700	2.73639700	1.69249900
N	-5.61148200	0.73529200	0.19013700
C	-6.84078000	1.07990100	1.00058500
H	-6.72568900	2.05507900	1.51738000
H	-7.74514400	1.12160000	0.37637700
H	-6.99775800	0.34247600	1.81077200
C	-5.85384300	-0.58004700	-0.51859700
H	-6.71482900	-0.52892600	-1.20054800
H	-4.96852600	-0.89101900	-1.11779000
H	-6.02317800	-1.40714900	0.19897300
O	-5.12699400	1.57779200	3.18644900
H	1.49151400	3.89461700	-0.56931300
Cl	-2.40260600	2.93829200	-0.08556800
H	-4.78395800	0.93944800	3.84520200
H	5.35954700	3.43785000	1.25461400
C	3.29050000	3.17193600	0.45370800
H	2.83009900	2.41721400	1.10149300
H	4.21591700	2.71605100	-1.66305600
H	1.77180800	1.59145300	-1.97142000
Zn	-0.38845700	-0.69958600	-0.85333600
Zn	-1.78535900	-1.83118000	-0.18105900
Cl	0.13550200	-3.23437800	-0.51024700
Cl	-1.20573500	-0.37313200	1.68840700
Cl	-2.53891800	-0.52066500	-2.15166600
Cl	-3.69547700	-2.84850400	0.43821700
O	0.30139500	-0.73423500	-2.86804000
H	-0.31164800	-0.71782200	-3.60607100
O	-0.26121000	1.29577900	-0.47487200
H	0.31380000	1.52918600	0.31472800
O	1.53336400	-1.30268100	2.31809900
H	0.73434200	-1.69940300	2.67702600

O	1.94719700	-2.74212500	-2.83055800
H	2.25852200	-2.57193400	-1.90812300
O	1.18378100	1.36373000	1.81607800
H	1.36250800	0.38800800	1.97629900
O	0.24586500	3.51757900	-1.69241600
H	0.08247600	2.53104200	-1.61672500
H	-0.60326300	3.93391900	-1.51002100
H	1.03452900	-1.40835600	-3.08817300
H	1.69013400	-1.73591500	1.46318400
H	1.34804600	-3.49751100	-2.76712800
H	0.53000500	1.60536800	2.47273800
H	-1.28193700	1.89559900	-0.23753500
C	-5.35641400	1.81166400	-0.84059300
H	-4.44410700	1.58059500	-1.43928100
H	-6.20153500	1.93365000	-1.53159300
H	-5.14185000	2.78968100	-0.36434400

Intermediate d) Int1

	(Hartree/Particle)
Zero-point correction=	0.515650
Thermal correction to Energy=	0.569886
Thermal correction to Enthalpy=	0.570830
Thermal correction to Gibbs Free Energy=	0.425004
Sum of electronic and zero-point Energies=	-0.564799
Sum of electronic and thermal Energies=	-0.510563
Sum of electronic and thermal Enthalpies=	-0.509619
Sum of electronic and thermal Free Energies=	-0.655445

	E (Thermal)	CV	S
	KCal/Mol	Cal/Mol-Kelvin	Cal/Mol-Kelvin
Total	357.609	191.003	306.916

Charge:0 Multiplicity: 1

C	2.91247800	0.09577200	-0.71460200
C	3.03350400	1.37503100	-1.17589600
C	3.96968600	2.17376700	-0.32802100
C	4.54412600	1.12408900	0.65883400
N	3.89738500	-0.11269300	0.37501000
O	5.35481400	1.31969300	1.53248800
O	2.12516400	-0.86989800	-1.00854900
C	4.25889600	-1.37566800	0.95019600
C	4.14659000	-2.55131600	0.18443500
C	4.71847200	-1.43163600	2.27948600
C	4.47795200	-3.77831900	0.76197700
H	3.81430300	-2.50182100	-0.85336800
C	5.04942200	-2.66770700	2.83506700
H	4.83879700	-0.51413300	2.86085200
C	4.92479100	-3.84268700	2.08514000
H	4.39373100	-4.69029100	0.17079000
H	5.40954200	-2.71451000	3.86308100
H	5.18339800	-4.80182700	2.52793300
N	1.74721000	3.22647300	0.24251000
C	1.31338500	4.38177000	-0.22387700
C	2.44147800	5.33148700	-0.34876300
H	0.26614600	4.59677300	-0.49682600
C	3.57228700	4.69796500	0.02961800
H	2.31420300	6.34114800	-0.70513900
C	-4.98010500	-0.71863600	1.41552600
C	-4.42706300	-0.54232100	2.83287700
H	-5.89788500	-1.34655200	1.43354500
H	-4.23221300	-1.30481300	0.80802100
H	-3.95173300	-1.48123000	3.17834300
H	-3.67701100	0.27374000	2.91906900
N	-5.28654600	0.59417900	0.69604000
C	-6.48989300	1.25220400	1.33549500
H	-6.35091700	1.32526800	2.44032700

H	-6.64716000	2.27249400	0.94943600
H	-7.41085100	0.67296400	1.16485000
C	-5.59331700	0.28855900	-0.75490200
H	-5.92324500	1.18685900	-1.30014500
H	-4.68627000	-0.09236900	-1.28724700
H	-6.36200700	-0.49383000	-0.86258100
O	-5.47320700	-0.13828800	3.72448100
H	1.16201100	2.25389800	0.31315700
Cl	-1.60658900	3.18100000	0.80290700
H	-5.96718600	-0.91296300	4.06098400
H	4.58387100	5.06951600	0.06469200
C	3.23709100	3.27813000	0.46665500
H	3.43452100	3.16718700	1.57259800
H	4.80606200	2.60799000	-0.92448000
H	2.51231600	1.79451100	-2.01256000
Zn	0.00571000	-0.49721700	-0.78291200
Zn	-1.66342600	-1.50833700	-0.98274700
Cl	0.17426100	-2.89956300	-1.68307700
Cl	-1.63442300	-1.02530000	1.40696800
Cl	-2.05800700	0.65186300	-2.02400300
Cl	-3.63830700	-2.57767900	-1.30884600
O	0.63566300	0.37304900	-2.74928400
H	0.05444000	0.24844900	-3.51607000
O	0.16950700	1.14245900	0.48017400
H	0.33649900	0.87171900	1.44787400
O	1.02959700	-1.91018100	0.94754100
H	0.54218300	-2.72635100	1.14721900
O	2.17714900	-1.50451800	-3.55110900
H	2.46682000	-1.71989200	-2.63033600
O	1.18810600	-0.01575400	2.58361300
H	1.88636100	-0.41508800	2.03192500
O	-0.30028400	3.24750500	-2.01073200
H	-0.00877500	2.35177900	-2.23824400
H	-1.22572300	3.17068500	-1.76282000

H	1.52819800	-0.05854100	-3.03821100
H	1.84017000	-2.20260100	0.44840800
H	1.48584800	-2.13992000	-3.75984800
H	0.54963900	-0.73743600	2.71514000
H	-0.79715600	1.95025300	0.58504100
C	-4.10906300	1.53835000	0.75580800
H	-3.17310400	1.03661400	0.42361000
H	-4.23869800	2.41738300	0.10105100
H	-3.91825800	1.91485800	1.77833600

Transition state d)TS2

	(Hartree/Particle)
Zero-point correction=	0.515213
Thermal correction to Energy=	0.568312
Thermal correction to Enthalpy=	0.569256
Thermal correction to Gibbs Free Energy=	0.426421
Sum of electronic and zero-point Energies=	-0.563238
Sum of electronic and thermal Energies=	-0.510139
Sum of electronic and thermal Enthalpies=	-0.509195
Sum of electronic and thermal Free Energies=	-0.652030

	E (Thermal)	CV	S
	KCal/Mol	Cal/Mol-Kelvin	Cal/Mol-Kelvin
Total	356.621	188.657	300.622

Charge:0 Multiplicity: 1

C	2.77561700	0.07239000	-0.88425300
C	2.63410900	1.24505200	-1.57630100
C	3.38141500	2.35456600	-0.90872900
C	4.24689700	1.61762500	0.14820600
N	3.83898800	0.24787400	0.13495900
O	5.07553100	2.10278100	0.88010000
O	2.14838100	-1.03809300	-0.90434100

C	4.45356600	-0.81289900	0.87699300
C	4.35385500	-2.14059400	0.41554800
C	5.14597900	-0.52956900	2.07004500
C	4.93147700	-3.17183600	1.15774700
H	3.84025800	-2.36505000	-0.52520600
C	5.72023000	-1.57447000	2.79358200
H	5.25066000	0.50238700	2.41615400
C	5.61101800	-2.89640300	2.34777600
H	4.85535700	-4.19873600	0.79991500
H	6.26006400	-1.35487600	3.71502800
H	6.06007200	-3.70444200	2.92032600
N	1.30364000	3.81614400	-0.99247900
C	1.02937200	5.06621400	-0.60564200
C	2.00937100	5.48368500	0.38299200
H	0.18905500	5.65380100	-0.97578200
C	2.86115800	4.43505700	0.60923800
H	2.02584200	6.46201200	0.83123300
C	-4.84067500	-0.22692600	1.64962700
C	-4.24693000	0.53745900	2.83698400
H	-5.74034700	-0.79794600	1.96866000
H	-4.09870800	-1.00629900	1.31532900
H	-3.72640000	-0.16046000	3.52167200
H	-3.52457700	1.32879100	2.53920400
N	-5.20632000	0.65711000	0.45781600
C	-6.40740500	1.50761200	0.81041800
H	-6.23478900	2.04147900	1.77501700
H	-6.60651900	2.26825400	0.03799500
H	-7.31604700	0.89868700	0.93786300
C	-5.54968800	-0.23521400	-0.71621200
H	-5.90217400	0.34793100	-1.58177800
H	-4.65445000	-0.80881800	-1.06269600
H	-6.31471700	-0.98568100	-0.45931500
O	-5.27744800	1.25827100	3.52312100
H	0.78028400	3.23537000	-1.67869000
Cl	-1.65591900	3.20936200	-0.59661100
H	-5.72894700	0.68832400	4.17788600
H	3.71751000	4.38459100	1.26926400
C	2.40158200	3.26710900	-0.17708200
H	1.80089000	2.49853300	0.69568200
H	4.03507500	2.92735700	-1.60349500

H	2.09805200	1.37480100	-2.49345400
Zn	-0.00253300	-1.00713300	-0.62415400
Zn	-1.62711500	-1.99255500	-0.30391200
Cl	0.30675100	-3.47841900	-0.34657300
Cl	-1.49713100	-0.45532000	1.60827300
Cl	-2.09123600	-0.46177200	-2.17397200
Cl	-3.57579700	-3.10811700	-0.05448900
O	0.58756800	-1.01349000	-2.80493300
H	0.05264900	-1.52772300	-3.42512700
O	-0.08283500	1.04544800	-0.39870100
H	0.49858000	1.47525700	-1.08853400
O	1.22427500	-1.20737000	1.35195400
H	0.81877000	-1.68118400	2.09545100
O	2.42215100	-2.83728100	-2.64201400
H	2.77619200	-2.54298300	-1.76213300
O	1.07862600	1.72286600	1.66167200
H	1.37387300	0.77581200	1.55369200
O	-0.12378100	1.93278100	-2.80706700
H	0.07514400	1.00773500	-3.06184500
H	-1.08795100	2.00292500	-2.77817100
H	1.53582800	-1.41979700	-2.87120300
H	2.05054200	-1.69510800	1.12673100
H	1.80918300	-3.55195800	-2.44450600
H	0.15458500	1.69666800	1.24866300
H	-1.01564900	1.74039400	-0.47483700
C	-4.05582400	1.55502200	0.06843300
H	-3.12422700	0.97037400	-0.09566500
H	-4.24694500	2.10703300	-0.86821800
H	-3.82459500	2.30722800	0.84758500

Intermediate b) Int2

	(Hartree/Particle)
Zero-point correction=	0.516850
Thermal correction to Energy=	0.571656
Thermal correction to Enthalpy=	0.572600
Thermal correction to Gibbs Free Energy=	0.425039

Sum of electronic and zero-point Energies= -0.602066
Sum of electronic and thermal Energies= -0.547261
Sum of electronic and thermal Enthalpies= -0.546317
Sum of electronic and thermal Free Energies= -0.693878

	E (Thermal)	CV	S
	KCal/Mol	Cal/Mol-Kelvin	Cal/Mol-Kelvin
Total	358.719	192.939	310.569

Charge:0 Multiplicity: 1

C	2.77490100	0.13393900	-0.86238600
C	2.59114700	1.28442500	-1.57645500
C	3.49485000	2.37531300	-1.07425900
C	4.40726400	1.64653500	-0.05981200
N	3.91617800	0.29277700	0.04523100
O	5.34528100	2.07431900	0.56132300
O	2.10993200	-0.96593800	-0.81210700
C	4.52314500	-0.75903900	0.80386600
C	4.33203600	-2.10347500	0.42875000
C	5.30339900	-0.44751000	1.93510500
C	4.90329400	-3.12143100	1.19453200
H	3.75568000	-2.35310500	-0.46822800
C	5.86995000	-1.47882400	2.68277100
H	5.48471900	0.59531300	2.21024400
C	5.66739100	-2.81664100	2.32391200
H	4.75586400	-4.16090600	0.90143700
H	6.47835000	-1.23688200	3.55474200
H	6.11153300	-3.61349900	2.91550400
N	1.53108900	3.96521000	-0.96134500
C	0.97520800	4.89146400	-0.07053800
C	1.80222900	4.96024400	1.04923400
H	0.05587600	5.40701100	-0.28462200
C	2.88611900	4.05626600	0.85008100

H	1.66121300	5.58377400	1.90866800
C	-4.81678900	-0.28800900	1.68402500
C	-4.21761600	0.52798300	2.83409900
H	-5.69505800	-0.86956600	2.04351900
H	-4.06683000	-1.06185900	1.35631100
H	-3.69072400	-0.13949100	3.54415400
H	-3.49858000	1.30772700	2.50256600
N	-5.23555500	0.54708300	0.47474300
C	-6.46997200	1.35080200	0.82303300
H	-6.31055500	1.91477600	1.77330700
H	-6.71546000	2.08236900	0.03609200
H	-7.34802400	0.70349300	0.97670800
C	-5.55314800	-0.38827600	-0.67311300
H	-6.03167700	0.14060600	-1.51217400
H	-4.62490100	-0.86050700	-1.07910100
H	-6.20501400	-1.22280100	-0.36387900
O	-5.24935600	1.27141600	3.49364200
H	1.14071100	3.69866300	-1.85283600
Cl	-1.94755200	3.27061200	-1.06330600
H	-5.66859900	0.74087100	4.20133100
H	3.71252700	3.90433100	1.51756300
C	2.70438900	3.44475700	-0.39264200
H	1.30385700	2.25961100	1.79050300
H	4.13140200	2.80887000	-1.88566600
H	1.95472300	1.41491200	-2.42744400
Zn	0.02505600	-0.96719300	-0.57963000
Zn	-1.59192800	-1.98341300	-0.30720200
Cl	0.35614500	-3.44092700	-0.34187100
Cl	-1.50009500	-0.49058800	1.63316000
Cl	-2.04292200	-0.39293000	-2.13199200
Cl	-3.53111200	-3.11867100	-0.11206000
O	0.61787500	-0.94545000	-2.77019100
H	0.09087800	-1.44457800	-3.40762900
O	-0.18739800	1.17824600	-0.42095600

H	0.66865100	1.64006500	-0.66561600
O	1.20280200	-1.17212200	1.45123000
H	0.84642300	-1.81098900	2.09066200
O	2.46018500	-2.73031100	-2.59837700
H	2.78065500	-2.45063700	-1.69686800
O	0.54954700	1.66608500	1.91155800
H	0.91054700	0.76638200	2.04927700
O	-0.24662400	1.88648200	-2.81122900
H	0.18664600	0.99224300	-2.79138400
H	-0.96241400	1.80635500	-3.45179000
H	1.57238200	-1.35167500	-2.83547300
H	2.09476000	-1.51561700	1.19410900
H	1.86415500	-3.46882000	-2.43981700
H	-0.33649600	1.51512900	0.56366000
H	-1.14957900	2.23085800	-1.44891600
C	-4.13606800	1.48880100	0.04116700
H	-3.16094000	0.96110600	-0.05854600
H	-4.34986500	1.94427600	-0.94227000
H	-3.97685000	2.31166000	0.76226000

Transition state b) TS3

	(Hartree/Particle)
Zero-point correction=	0.513692
Thermal correction to Energy=	0.567663
Thermal correction to Enthalpy=	0.568608
Thermal correction to Gibbs Free Energy=	0.423048
Sum of electronic and zero-point Energies=	-0.595689
Sum of electronic and thermal Energies=	-0.541717
Sum of electronic and thermal Enthalpies=	-0.540773
Sum of electronic and thermal Free Energies=	-0.686333

	E (Thermal)	CV	S
	KCal/Mol	Cal/Mol-Kelvin	Cal/Mol-Kelvin
Total	356.214	190.813	306.356

Charge:0 Multiplicity: 1

C	2.75971700	-0.08724300	-0.74882300
C	2.50058500	1.14619800	-1.40252200
C	3.59526600	2.13405300	-1.07470800
C	4.50550900	1.37043300	-0.09106600
N	3.96284800	0.01411600	0.02527800
O	5.49871300	1.71883100	0.48254800
O	2.05609700	-1.13632900	-0.70511100
C	4.51885500	-1.04949600	0.81602400
C	4.26898900	-2.38813600	0.45886200
C	5.30494000	-0.74997800	1.94419900
C	4.79807100	-3.41732400	1.24081700
H	3.68533300	-2.62649800	-0.43517500
C	5.82760500	-1.79269400	2.70869000
H	5.51910900	0.28992800	2.20912800
C	5.57269900	-3.12606300	2.36652800
H	4.60970800	-4.45435500	0.96340200
H	6.44119900	-1.56292600	3.58059000
H	5.98410600	-3.93173000	2.97065000
N	1.86014000	3.96599900	-0.82293700
C	1.71965700	5.18331000	-0.15339100
C	2.88504600	5.41037000	0.58242900
H	0.83061800	5.78067700	-0.23512200
C	3.75881700	4.30911300	0.36542700
H	3.09070900	6.25775700	1.20267900
C	-5.02257800	-0.13980000	1.54082300
C	-4.44897500	0.51498000	2.80160000
H	-5.95542200	-0.69515900	1.78464200
H	-4.29685100	-0.92333100	1.18208400
H	-4.00750300	-0.25514800	3.46428300
H	-3.66721200	1.27532900	2.58846500
N	-5.30704100	0.83733400	0.40103000
C	-6.49011000	1.70800000	0.76482800
H	-6.33927300	2.16957900	1.76982300
H	-6.63086600	2.52604200	0.03923100
H	-7.42495300	1.12758300	0.81444600

C	-5.63460700	0.04222300	-0.84542400
H	-5.96606500	0.69400200	-1.66966000
H	-4.73938700	-0.51306700	-1.21821800
H	-6.41244400	-0.71758900	-0.66275100
O	-5.46705000	1.25605800	3.48244600
H	1.16788700	3.53933200	-1.42775900
Cl	-1.63570200	3.29093700	-0.47729600
H	-5.98488500	0.67783400	4.07943100
H	4.73413800	4.17843900	0.79208500
C	3.11117000	3.42060400	-0.49956100
H	0.29220100	1.41552800	0.73287800
H	4.21100200	2.35929200	-1.98958000
H	2.04082800	1.18310700	-2.38498600
Zn	-0.13592700	-0.87067700	-0.57210100
Zn	-1.79451400	-1.85141300	-0.43521000
Cl	0.07215400	-3.40207200	-0.60967300
Cl	-1.65671500	-0.54611400	1.64834400
Cl	-2.09392200	-0.14969900	-2.17085900
Cl	-3.78794000	-2.90199400	-0.34963900
O	0.57270400	-0.95744800	-2.70151500
H	-0.00247200	-1.34749400	-3.37401400
O	0.13213600	1.15954500	-0.24905700
H	1.20900500	1.47992800	-0.78115200
O	0.98290500	-1.53067800	1.43679900
H	0.57401300	-2.26762800	1.92005900
O	2.33779500	-2.89143300	-2.54688300
H	2.57823700	-2.73286200	-1.60042300
O	0.71794400	1.12612700	2.30892300
H	0.90690800	0.16251500	2.29260800
O	-0.08098600	2.11909400	-2.52381000
H	0.13141600	1.20627100	-2.78346500
H	-0.94889500	2.31175100	-2.88516900
H	1.44539200	-1.51003400	-2.74477600
H	1.87600100	-1.85259200	1.15737300
H	1.74544000	-3.64957700	-2.54526600
H	-0.09113900	1.22884300	2.81348900
H	-0.77225100	2.19723100	-0.66988600
C	-4.10976900	1.71443100	0.11853800
H	-3.18053900	1.11273400	0.00661100
H	-4.22302900	2.28772900	-0.81878200

H	-3.91970900	2.44308200	0.92873100

N-Phenyl succinimide + ChCl + 2 $ZnCl_2$ • $3H_2O$ d)P

	(Hartree/Particle)
Zero-point correction=	0.516965
Thermal correction to Energy=	0.571622
Thermal correction to Enthalpy=	0.572566
Thermal correction to Gibbs Free Energy=	0.423305
Sum of electronic and zero-point Energies=	-0.620928
Sum of electronic and thermal Energies=	-0.566271
Sum of electronic and thermal Enthalpies=	-0.565327
Sum of electronic and thermal Free Energies=	-0.714588

	E (Thermal)	CV	S
	KCal/Mol	Cal/Mol-Kelvin	Cal/Mol-Kelvin
Total	358.698	190.752	314.146

Charge:0 Multiplicity: 1

C	3.59611200	-0.41475300	-1.06561700
C	3.77234000	0.98888000	-1.57740600
C	4.47719200	1.78136000	-0.45924700
C	4.73645700	0.76611900	0.66812600
N	4.15929800	-0.50432100	0.23858900
O	5.31222400	0.90178200	1.70725200
O	3.05382100	-1.35211300	-1.62996000
C	4.14139400	-1.70308100	1.04609200
C	4.24837600	-2.96084600	0.42799600
C	4.00252500	-1.59513800	2.44035200
C	4.21504800	-4.11168800	1.21855100
H	4.36353600	-3.04202000	-0.65399700
C	3.97632900	-2.75808400	3.21199300
H	3.92781100	-0.61484100	2.92025200
C	4.07853700	-4.01542700	2.60672100
H	4.30287500	-5.09022900	0.74621900

H	3.87563900	-2.67971300	4.29523500
H	4.05880700	-4.91665200	3.21668800
N	2.38240900	2.84371400	0.53134300
C	1.98128900	4.10902300	0.95830900
C	3.03472800	5.00193900	0.73814800
H	1.01391600	4.29343900	1.39934000
C	4.11154400	4.27058900	0.17089700
H	3.03524200	6.04952600	0.96121900
C	-4.97590200	-0.65099500	1.43718800
C	-4.31362000	-0.73388100	2.81646800
H	-5.94497200	-1.19787700	1.44215300
H	-4.33040800	-1.20091200	0.69532000
H	-3.93297800	-1.75888700	2.99498500
H	-3.46762400	-0.02551700	2.94562100
N	-5.21047700	0.77415800	0.93787400
C	-6.30940200	1.42273400	1.75129500
H	-6.09272500	1.32834500	2.84214500
H	-6.40271500	2.49728600	1.52111000
H	-7.28646400	0.94676900	1.57372100
C	-5.63704800	0.70651000	-0.51380000
H	-5.89117800	1.70323600	-0.91062500
H	-4.81698200	0.30849000	-1.15966100
H	-6.50127800	0.03902200	-0.66308000
O	-5.24119900	-0.34737400	3.83595400
H	1.80477600	1.99672300	0.55481200
Cl	-1.37860900	3.14096500	1.00477400
H	-5.79756900	-1.10443100	4.11166000
H	5.06553300	4.67001200	-0.10503400
C	3.69717700	2.93747400	0.04962900
H	5.47435300	2.15380100	-0.81921600
H	2.76633800	1.41974000	-1.83621700
Zn	-0.33178700	-0.30671300	-0.78436400
Zn	-1.91416100	-1.27608900	-1.30620700
Cl	-0.03869900	-2.24077600	-2.46685300

Cl	-1.65246400	-1.43020500	1.17102300
Cl	-2.21696700	1.03491300	-1.95799500
Cl	-3.91586900	-2.24681700	-1.63269400
O	0.76704500	0.94915600	-2.04702800
H	0.40068600	1.92303700	-2.05487900
O	0.21305000	0.93793100	0.67611900
H	0.33512900	0.56395900	1.59270900
O	1.02838900	-1.92385400	0.13730900
H	0.58474000	-2.78938200	0.24255500
O	1.69705700	-0.37305100	-3.98561400
H	2.14871200	-0.92180100	-3.30627300
O	0.96953700	-0.67038400	2.62758700
H	1.34323900	-1.20007400	1.89375200
O	-0.17038300	3.39266800	-1.76797200
H	0.28851600	3.75655900	-0.99379300
H	-1.10188600	3.31777300	-1.50122000
H	0.83956000	0.68153100	-3.02560900
H	1.82454600	-2.12380200	-0.43796700
H	1.08593200	-0.96056900	-4.44028700
H	0.21639500	-1.16597600	2.95246900
H	-0.51192900	1.91199700	0.86094000
C	-3.95507000	1.60867000	1.04235400
H	-3.06480600	1.05672800	0.67564300
H	-4.01254200	2.53268300	0.43696000
H	-3.73091200	1.91759400	2.08022600
H	4.33047300	1.01177100	-2.53321100
